# The influence of temperature on cellulose swelling at constant water density

**DOI:** 10.1038/s41598-022-22092-5

**Published:** 2022-12-01

**Authors:** Jonathan Torstensen, Vegar Ottesen, Sandra Rodríguez-Fabià, Kristin Syverud, Lars Johansson, Anders Lervik

**Affiliations:** 1 Western Norway University of Applied Sciences, Bergen, Norway; 2 Department of Chemical Engineering, NTNU, Trondheim, Norway; 3 RISE PFI, Trondheim, Norway; 4 Department of Chemistry, NTNU, Trondheim, Norway

**Keywords:** Chemistry, Energy science and technology, Engineering, Materials science, Nanoscience and technology

## Abstract

We have in this paper investigated how water sorbs to cellulose. We found that both cellulose nanofibril (CNF) and cellulose nanocrystal (CNC) films swell similarly, as they are both mainly composed of cellulose. CNF/CNC films subjected to water at 0.018 kg/m^3^ at 25 °C and 39 °C, showed a decrease in swelling from ~ 8 to 2%. This deswelling increased the tensile index of CNF-films by ~ 13%. By molecular modeling of fibril swelling, we found that water sorbed to cellulose exhibits a decreased diffusion constant compared to bulk water. We quantified this change and showed that diffusion of sorbed water displays less dependency on swelling temperature compared to bulk water diffusion. To our knowledge, this has not previously been demonstrated by molecular modeling. The difference between bulk water diffusion (D_WW_) and diffusion of water sorbed to cellulose (D_CC_) increased from D_WW_ − D_CC_ ~ 3 × 10^–5^ cm/s^2^ at 25 °C to D_WW_ − D_CC_ ~ 8.3 × 10^–5^ cm/s^2^ at 100 °C. Moreover, water molecules spent less successive time sorbed to a fibril at higher temperatures.

## Introduction

Nanocelluloses are cellulosic nanomaterials (CNMs) with at least one dimension on the nanometer scale (below 100 nm). They are rod- or ribbon-shaped nanoparticles made from multiple cellulose polymer chains, with typical widths below 10 nm and lengths in the nm–µm region. The ensemble of cellulose chains forms an intricate amorphous/crystalline bundle structure which is highly debated^1,2^. Nanocelluloses display properties characteristic of both cellulose and nanomaterials^3^. Some of the main features of nanocelluloses are their hydrophilicity, multifunctionality and potential for chemical modification. Moreover, nanocelluloses have a large surface area, are biocompatible, and are biodegradable^4,5^. Depending on the cellulosic source and production method, nanocelluloses may be classified into cellulose nanocrystals (CNCs), cellulose nanofibrils (CNFs), tunicate nanocellulose, or bacterial nanocelluloses (BNCs)^3^. CNCs are typically obtained from hydrolysis of cellulose fibers^3,6^. Conversely, CNFs are produced by extracting nanocellulose from the plant cell wall by various methods, whereas BNCs are obtained from bacteria. Nanocelluloses from different sources or made by different methods may vary in degree of crystallinity, surface chemistry and aspect ratios^7^.

CNF and CNC films differ in mechanical properties due to the morphology and dimensions of their nanostructures. While CNFs typically have large aspect ratios and form entanglements, CNCs predominantly consist of shorter, rod-like bundles with high crystallinity. These differences affect the properties of CNF and CNC films. CNF films typically have an elastic modulus of 10 GPa and a strength of 100 MPa, although they can reach 20 GPa and 240 MPa, respectively^8^. On the other hand, CNC films have fewer entanglements and thus have poor mechanical properties and low toughness. Bras et al.^9^ investigated the tensile strength of a series of CNC films from various sources and reported values between 0.4 and 11 GPa. The tensile moduli seemed to increase with the increasing aspect ratio of the CNCs. However, other factors, such as the films' porosity, density, or the crystals' alignment, can influence their mechanical properties. The mechanical properties of CNC^10,11^ or CNF^12^ films can be improved by chemical modification or decreasing crystallinity.

Water swelling has a negative impact on cellulosic material applications in packaging^13,14^ and as composite additives^15^. It is thus important to investigate such behaviour in neat cellulosic materials, to better be able to tune or reduce water swelling.

Studies in this paper were performed with constant water present, and varying temperature. This was done to highlight the importance of temperature in cellulosic material swelling. This paper briefly summarizes relevant experimental work on cellulose water swelling. We then investigate cellulose film water vapour swelling in CNF/CNC films. Then, molecular dynamic simulations is done to describe the diffusion of water molecules sorbed to a nanocellulose.

## Theoretical background

### Water vapour sorption of a cellulosic film

**Figure 1:**
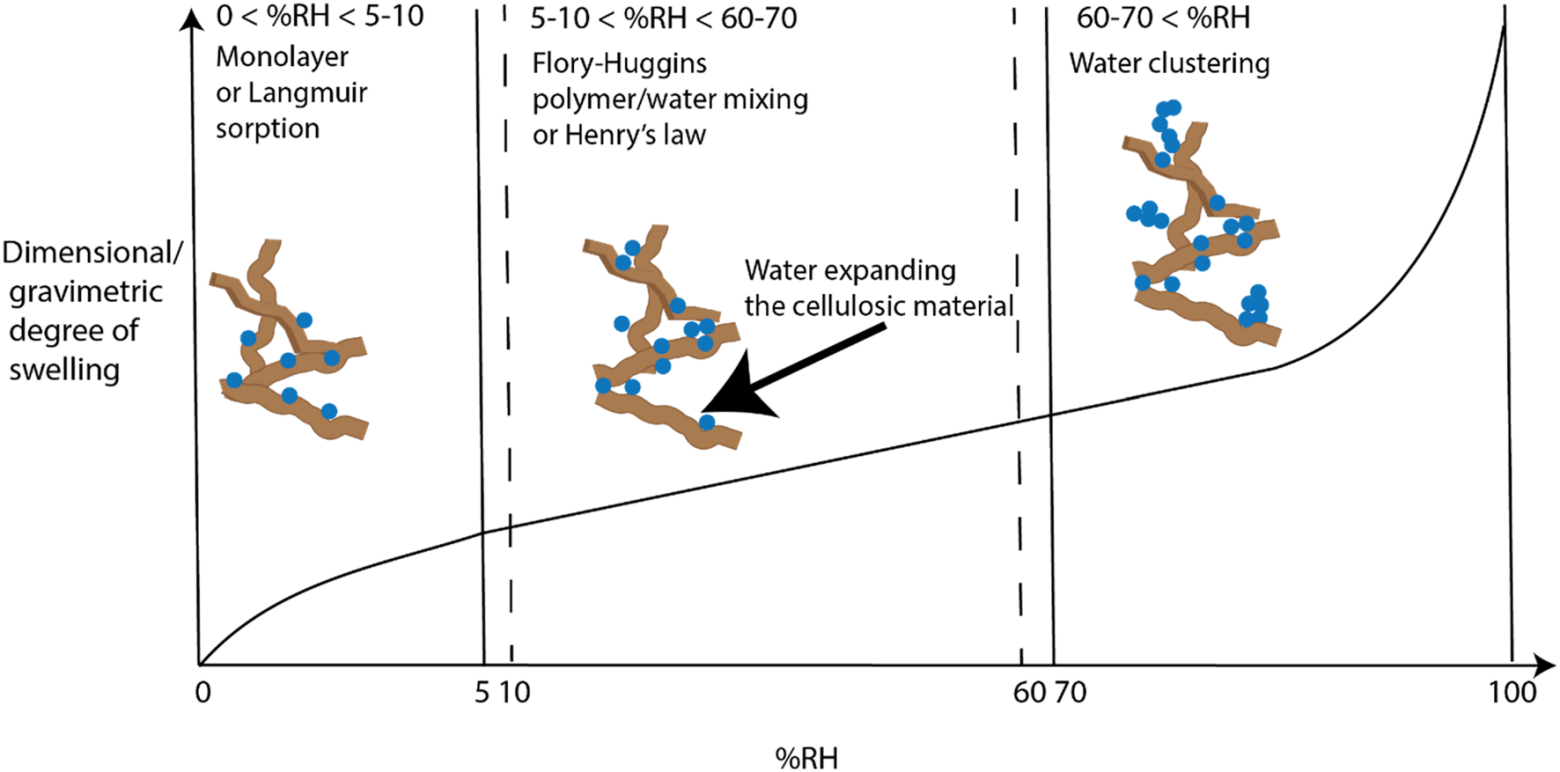
Illustration of water sorption models as described by Belbekhouche et al.^17^ (for the MFC type of nanocellulose) and Hakalahti et al., for TEMPO-oxidized CNF^16^.

Water vapour sorption models have been investigated by Hakalahti et al. for TEMPO-oxidized CNF films^16^, and with dynamic water vapour sorption by Belkebouche et al.^17^, for mechanically fibrillated CNF films. They observed three distinct types of water sorption and found that the Park model was the best fit for the adsorption isotherm. The Park model has three sorption modes: Langmuir monolayer sorption, Henry's law type sorption, and water clustering. Water vapour sorption is expressed as a function of the water activity, a = %RH/100%. For mechanically fibrillated cellulose, the three different sorption phases were observed: The initial low humidity Langmuir monolayer sorption, where the first monolayer of water is sorbed onto cellulose, was found to be dominating at water vapour activity, a < 0.1. In the second phase, from 0.1 < a < 0.6, Henry's law was representative of the swelling, e. g. a linear increase of film water content with relative humidity. Finally, from a > 0.6, clustering dominated swelling. A very similar model was later devised by Hakalahti et al.^16^ for TEMPO-oxidized CNF films. Langmuir (water monolayer) sorption was most prominent at a < 0.05. The second phase, ending at a = 0.6, was described by a Flory–Huggins approach. While Henry's law interpretation is seemingly fair in this relative humidity regime, it does not account for conformational changes in the polymer during swelling. Polymers generally undergo conformational changes upon swelling, known from Flory–Huggins mixing theory. Hakalahti et al.^16^ devised an expression for the water sorbed in the Flory Huggins regime (approximately the same as the Henry regime in Belbekhouche et al.^17^). For activities above a = 0.6, Hakalahti et al. applied a clustering model with a clustering term. In both studies, the water activity is the relative humidity fraction. The sorption models devised by Belbekhouche et al.^17^ and Hakalahti et al.^16^ are summarized in Fig. 1 and Table 1.

**Table 1:** Water sorption model—expressions as described by Belbekhouche et al.^17^ for the MFC type of nanocellulose and Hakalahti et al., for TEMPO-oxidized CNF^16^.

Sorption type	Expression	%RH regime
Belbekhouce et al.^17^ Langmuir capacity constant, A_L_ = 1 Langmuir affinity constant, B_L_ = 200 Henry's solubility coefficient, K_H_ = 13.7 Equilibrium constant for water clustering, K_C_ = 18.2 Number of water molecules in a cluster, n = 11 Water activity, a (= %RH/100%)		
Langmuir (monolayer) fraction, F_L_	${\mathrm{F}}_{\mathrm{L}}=\frac{ {\mathrm{A}}_{\mathrm{L}}{\mathrm{B}}_{\mathrm{L}}\mathrm{a}}{ 1+{\mathrm{B}}_{\mathrm{L}}\mathrm{a}}$	< 10
Henry's law fraction, F_H_	${\mathrm{F}}_{\mathrm{H}}= {\mathrm{K}}_{\mathrm{H}}\mathrm{a}$	10–60
Water clustering fraction, F_C_	${\mathrm{F}}_{\mathrm{C}}= {\mathrm{K}}_{\mathrm{C}}{\mathrm{na}}^{\mathrm{n}}$	> 60
Hakalahti et al.^16^ Concentration of specific sorption sites, A_L_ = 1.41 Concentration of specific sorption sites, B_L_ = 223 The Flory–Huggins interaction parameter, χ = 0.67 Equilibrium constant for water clustering, K_C_ (not stated) Number of water molecules in a cluster, n = 10 Water activity, a (= %RH/100%)		
Langmuir (monolayer) fraction, F_L_	${\mathrm{F}}_{\mathrm{L}}= \frac{{\mathrm{A}}_{\mathrm{L}}\mathrm{a}}{1+{\mathrm{B}}_{\mathrm{L}}\mathrm{a}}$	< 5
Flory–Huggins fraction, F_FH_	$\mathrm{ln}\left(\mathrm{a}\right)=\mathrm{ln}\left({\mathrm{F}}_{\mathrm{FH}}\right)+\left(1-{\mathrm{F}}_{\mathrm{FH}}\right)+\mathrm{\rm X}{\left(1-{\mathrm{F}}_{\mathrm{FH}}\right)}^{2}$	30–70
Water clustering, F_C_	${\mathrm{F}}_{\mathrm{C}}={\mathrm{K}}_{\mathrm{c}}{\mathrm{a}}^{\mathrm{n}}$	> 70

Other relevant sorption models are discussed by Belbekhouche et al.^17^. Another possible model is the GAB swelling model. This model describes sorption as first monolayer then multilayer sorption. The GAB and Park model give similar sorption isotherms. However, they may also yield material-dependent differences. At present, it is not evident which model is more physically accurate^17,18^. The degree of crystallinity in nanocellulose films has also been found to affect swelling. Highly amorphous cellulose films were compared to 60% crystalline cellulose films^19^. Swelling was nearly identical in these films up to 75%RH at 23 °C. However, the difference between crystalline and amorphous cellulose was more pronounced at 97%RH. The amorphous cellulose films swelled 33.4%, and the 60% crystalline films swelled 45.8%. Other works^20^ find a more noticeable difference between CNF/CNC films at lower relative humidities and attribute increased CNF film swelling to their more amorphous structure.

### Liquid water sorption of a native fibril or cellulosic fiber

In a very instructive paper written by Grignon et al.^21^, the swelling of a (nano) cellulose surface was modeled. They showed that the distribution of ions and water in the surface and gel region surrounding the fibril differs from that of the bulk. An asymmetric ion distribution between the gel and bulk and cellulose-water interactions were considered the driving forces of surface water (ad)sorption. Ottesen et al. quantized fibril liquid water swelling by atomic force microscopy, finding degrees of swelling around 30–40%^22^. Seemingly, these values are similar to amorphous cellulose liquid water swelling, e. g. 35% found by Esker et al.^23^ The principle difference between CNFs and CNCs, is crystallinity if one disregards surface charge- and type. Crystalline cellulose is impregnable to water, used as an argument to characterize CNC water sorption as only adsorption and not absorption^24^. However, most CNC types are not entirely crystalline, and most CNF types contain crystallites^7^. We thus reason that one possible adaption would be incorporating the degree of crystallinity in the applied sorption models.

## Experimental

### Materials

#### Cellulose nanocrystals (CNCs)

Cellulose nanocrystals were purchased from the University of Maine USDA Forest Products Laboratory and were of the sulfate ester (sulfate hydrolysis process) type (~ 11–12 wt% slurry)^25^. This nanocellulose is subsequently referred to as CNC in the remainder of this paper.

#### Cellulose nanofibrils (CNFs)

The preparation of cellulose nanofibrils is described by Ottesen et al.^12^. Briefly, two types of CNFs were prepared from cotton linters (Celsur, Spain). One type had a high degree of crystallinity (CNF-H), while one had a low degree of crystallinity (CNF-L). The degree of crystallinity was modified before fibrillation. Reduction in the degree of crystallinity was achieved by ammonia treatment followed by boiling in water. Linters were submerged in liquid anhydrous ammonia (99.98%, R717, AGA, Oslo) at atmospheric pressure for 8 h. After 8 h had elapsed, cooling was stopped, and ammonia could evaporate overnight. After ammonia evaporation, the treated linters were boiled in de-ionized water for 5 h before drying in air at 100 °C overnight. CNFs with a low degree of crystallinity (CNF-L) were treated thrice in this manner before mechanical treatment. CNFs with a high degree of crystallinity (CNF-H) were not ammonia-treated but were boiled for 5 h and then dried overnight at 100 °C. The chemically treated linters were then beaten at 10,000 revolutions in a PFI mill (Hamjern Maskin, Hamar, Norway) at 10 wt% solid content. After beating the linters, they were passed through a Masuko supermasscolloider (Masuko, Japan) at 1 wt% solid content. Grinding was performed by 12 passes at 2000 RPM. After grinding the linters, now slightly below 1 wt% solids, they were homogenized using a Rannie 15 type 12.56 × homogenizer (APV, SPX Flow Technology, Silkeborg, Denmark) 5 times. The first pass was done at a 600 bar pressure drop, and all subsequent passes were performed at 1000 bar.

### Suspension characterization

#### Suspension macroscopic morphology characterization

An L&W Fibertester PLUS (Kista, Sweden) was used to investigate the macroscopic sample morphology. Three parallels (3 × 0.1 g) were run per sample type (CNC, CNF-L, and CNF-H). Fines were defined as constructs with lengths > 7 µm and < 200 µm (lower detection limit supplied by the manufacturer). Objects are constructs with widths between 75 µm and 10 000 µm and lengths between 100 µm and 10 000 µm. Reported errors are standard errors.

#### Suspension nanoscopic morphology characterization

Suspension nanoscopic morphology was characterized by atomic force microscopy (AFM). Samples for AFM investigation were prepared in the following manner: CNF suspensions with a concentration of 0.02 wt% were prepared and sonicated for 2 min with an Elmasonic P 30 H (Singen, Germany), 37 kHz at 100% power. One droplet of the fibril suspension was then deposited onto a flat substrate. Most of the water was wicked away using a non-linting paper, and the sample was baked for 20 min at 85 °C. The substrate used for AFM samples was mica (grade V1 muscovite, Ted Pella, Redding, CA, USA). The mica was freshly cleaved and had been plasma treated (O_2_, 1 min) to activate the surface before droplet addition.

Images were acquired on a Veeco Multimode V AFM (NY, USA), using Nanoscope 8.15 software using an E-scanner (s/n 10054EVLR). Micrographs were recorded using Bruker's proprietary ScanAsyst tapping-mode. Scan rate was set to 0.888 Hz (CNF-L) and 0.977 Hz (CNF-H). At 1024 × 1024 pixels. The tip used was a Bruker ScanAsyst Fluid tip, reported to have a nominal tip radius of 20 nm and a maximal tip radius of^26^ 60 nm.

### Nanocellulose film fabrication and characterization

#### Film preparation

Films were prepared by solvent casting 50 g of 0.5 wt% suspensions in Petri dishes (circular, diameter: 9 cm, height: 1.5 cm). Films were ambiently dried and stored in a desiccator for more than five days before swelling and mechanical characterization.

#### Thermogravimetric analysis and differential scanning calorimetry

Thermogravimetric Analysis (TGA) and Differential Scanning Calorimetry (DSC) were run using a NETSCH STA 449F3 (Selb, Germany). About 10–20 mg sample was used, and two parallels were run per sample. Samples were desiccated for > 5 days before the analysis. Specimens were then analyzed in the range of 30 °C to 800 or 1000 °C, with heating of 10 °C/min. Presented curves are the average of two interpolated sample curves. TGA and DSC were performed in 100% nitrogen (99.999%, Linde, Oslo, Norway) or atmospheric air.

#### Film relative humidity swelling

Relative humidity (RH) swelling tests were performed on films cast from 0.5 wt% suspensions. Swelling trials were conducted at two different temperatures and relative humidities. One series was subjected to 25 °C, and 80%RH (state A), and the same film specimens were then subjected to 39 °C and 36%RH (state B). Films were kept in the environmental chamber between state transitions. The temperature and RH uncertainties were +/− 0.3 °C and +/− 0.4%RH. State A corresponds to a vapor pressure of 2.52 kPa (3.169 kPa × 0.8)^27^. State B corresponds to a vapour pressure of 2.54 kPa (6.9969 kPa × 0.36)^27^. This corresponds to ~ 0.018 kg/m^3^ water, calculated by the method of Wagner et al.^28^. States A and B were chosen to obtain the largest difference in temperature while retaining the same absolute humidity/water vapour partial pressure. Swelling tests were performed in a Termaks Environmental Chamber (Bergen, Norway). Tests were done with four or five replicates. The swelling, S, was calculated as.

1 $$\mathrm{S}=\left(\frac{{\mathrm{W}}_{\mathrm{t}}}{{\mathrm{W}}_{\mathrm{O}}}-1\right)\times 100\mathrm{\%},$$

where W_t_ is the weight at t hours after swelling initiated, and W_o_ is the dry weight. The reported errors are standard errors.

#### Film mechanical testing

Films were cut into square test specimens and swollen at State A and then State B as described above. Mechanical testing was performed on a Zwick/Roell ZMART.PRO (Ulm, Germany), employing the ISO 1924-3 Standard^29^ with a force of 2.5 kN. At least four parallels were run per sample type.

### Molecular modeling

#### Nanocellulose models

Our model's cellulose molecules were built from a cellobiose template (ATB molecular ID 32,442)^30–32^. This template was used to construct a cellulose chain consisting of 52 glucose units (25 cellobiose units + two end glucose residues), ca. 26.9 nm in length from the first C4 to the last C1 atom. We recognize that glucose is the repeating unit in cellulose^33^. However, cellobiose was found to be the most appropriate starting point for our model, as it has incorporated the 180° rotation of the C6 OH-group along the screw-axis of cellulose.

**Figure 2:**
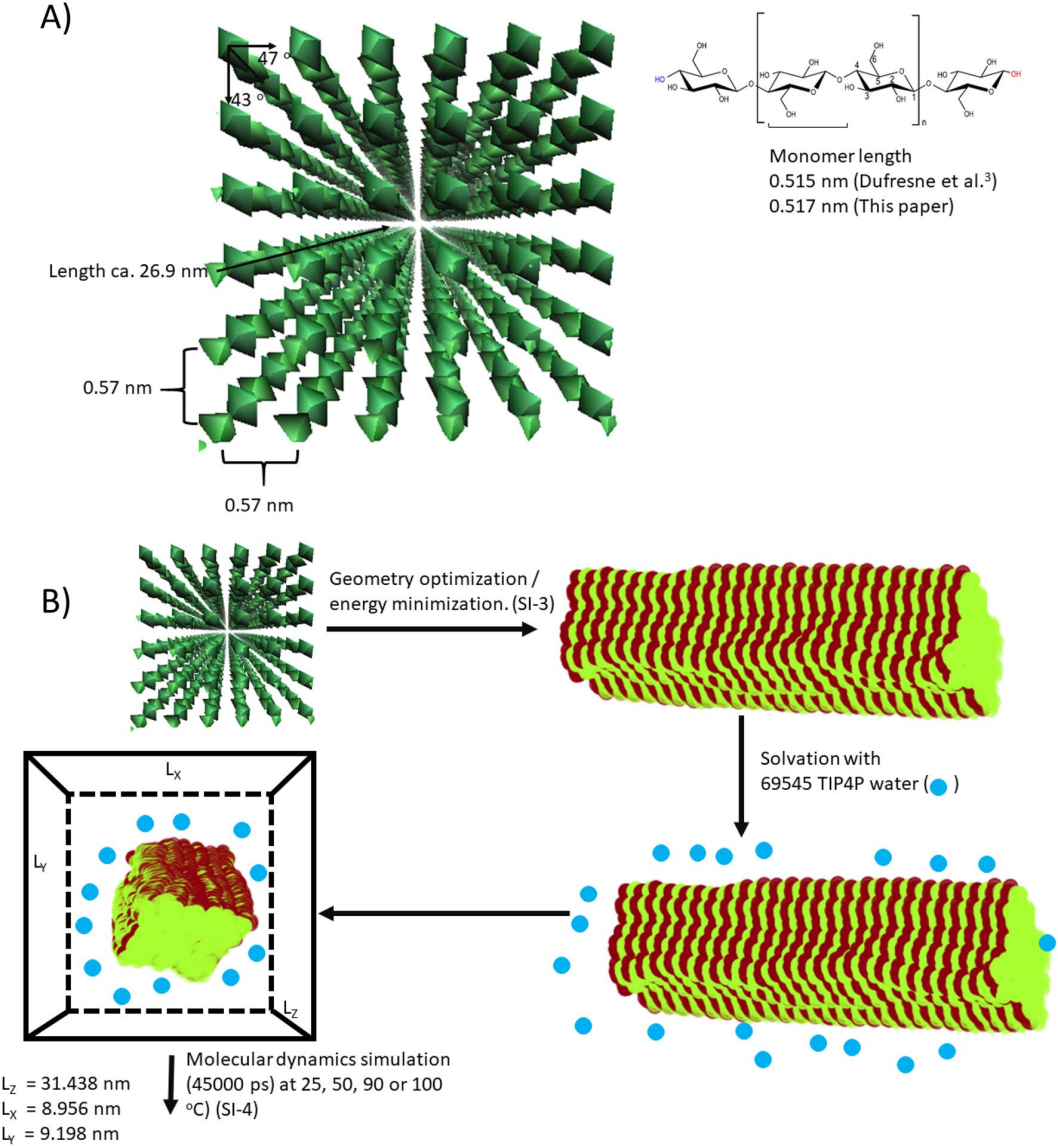
Overview of the initial cellulose bundle/nanofibril and modeling in this paper. (**A**) The fibril structure and (**B**) the performed modeling.

The cellulose elementary fibril was modeled inspired by Sèbe et al.^34^. A grid was made of 6 × 6 chains (36 in total). We chose a 36-chain system instead of the most probable 18-chain configuration^35–37^. to provide a larger system. A larger cellulose structure provides a larger surface and bulk. It also facilitates data interpretation and thus provides a better model for fundamental cellulose-water interactions. The model gives 6 + 6 + 4 + 4 = 20 surface chains, or a surface fraction of 20/36 = 0.56 (Fig. 2).

Chains were positioned 0.57 nm apart (from Fig. 10 in Sèbe et al.^34^). The chain unit vector was calculated from the first C4 to the last C1. This vector was used to rotate the chains, approximating the shape in Fig. 8b in Sèbe et al.^34^, where chains make 43° and 47° with either the x- or y-axis (Fig. 2). Overview of simulations is given in Fig. 2B. The initial configuration for the fibril was created using Maestro^38^ and exported to GROMACS, version 5.1.4^39^. This initial configuration was geometry optimized in GROMACS using a steepest-decent energy minimizer. The interactions were modeled using the OPLS-AA force field^40^, where we obtained Coulombic forces using the smooth particle mesh Ewald (PME) method (with a real-space cut-off of 1 nm)^41^. The van der Waals interactions were truncated at 1 nm, and dispersion corrections were applied to the energy and pressure terms. We applied periodic boundary conditions in all directions, and all bonds were constrained with the LINCS algorithm^42^. After the energy optimization, the fibril was solvated in water using the TIP4P force field^43^. For all simulations, 69,545 water molecules were used. The box had dimensions L_x_ = 8.956, L_y_ = 9.198 and L_z_ = 31.438 nm. These dimensions were chosen to fit the water molecules and bundle and to ensure more than 1 nm (the cut-off) space around the bundle. The chosen dimensions corresponded to a box volume of V_box_ = 2590 nm^3^. The water density was 803 kg/m^3^ (= ((69,545/6.022 × 10^23^ mol^−1^)/2590 nm^3^) × 18 × 10^–3^ kg mol^-1^ × 10^27^ nm^3^/m^3^). At all temperatures, the water density was lower than the TIP4P water density, which was above 900 kg/m^3^ in the entire investigated temperature regime^44^. Following a geometry optimization of the solvated system, we performed molecular dynamics (MD) simulations at the target temperatures (25, 50, 90, or 100 °C). For these simulations, the temperature was controlled using the thermostat of Bussi et al.^45^, with a time-constant of 0.5 ps. The simulation time step was 0.1 ps, and simulations lasted 45 ns (45,000 ps). Data were recorded with a time-step of 10 ps.

#### Modeling data analysis

For all analyses, carbon atoms were used to represent the fibril construct, while oxygen atoms represented water. Only oxygen atoms were used to be computationally able to analyze all timeframes.

##### Nearest neighbor (NN) analysis

The default algorithm in Matlab (knnsearch) applies the Euclidean distance to locate the 50 nearest oxygen neighbors of each carbon atom. These were used to determine the nearest neighbour distance between water and cellulose during swelling.

##### Fibril volume

The fibril volume was calculated using the convhull (convex hull) Matlab function on the carbon atom point cloud.

##### Delaunay triangulation

The delaunayTriangulation Matlab function was applied to represent the fibril. Together with pointLocation it was used to determine the position of water molecule oxygen atoms relative to the fibril, i. e. sorbed to or in the bulk. For molecular modeling, the pointLocation function was used to determine W_t_ in Eq. 1.

## Results

### Part I: Experimental characterization of nanocellulose and nanocellulose film swelling

#### Size, morphology, and charge

**Figure 3:**
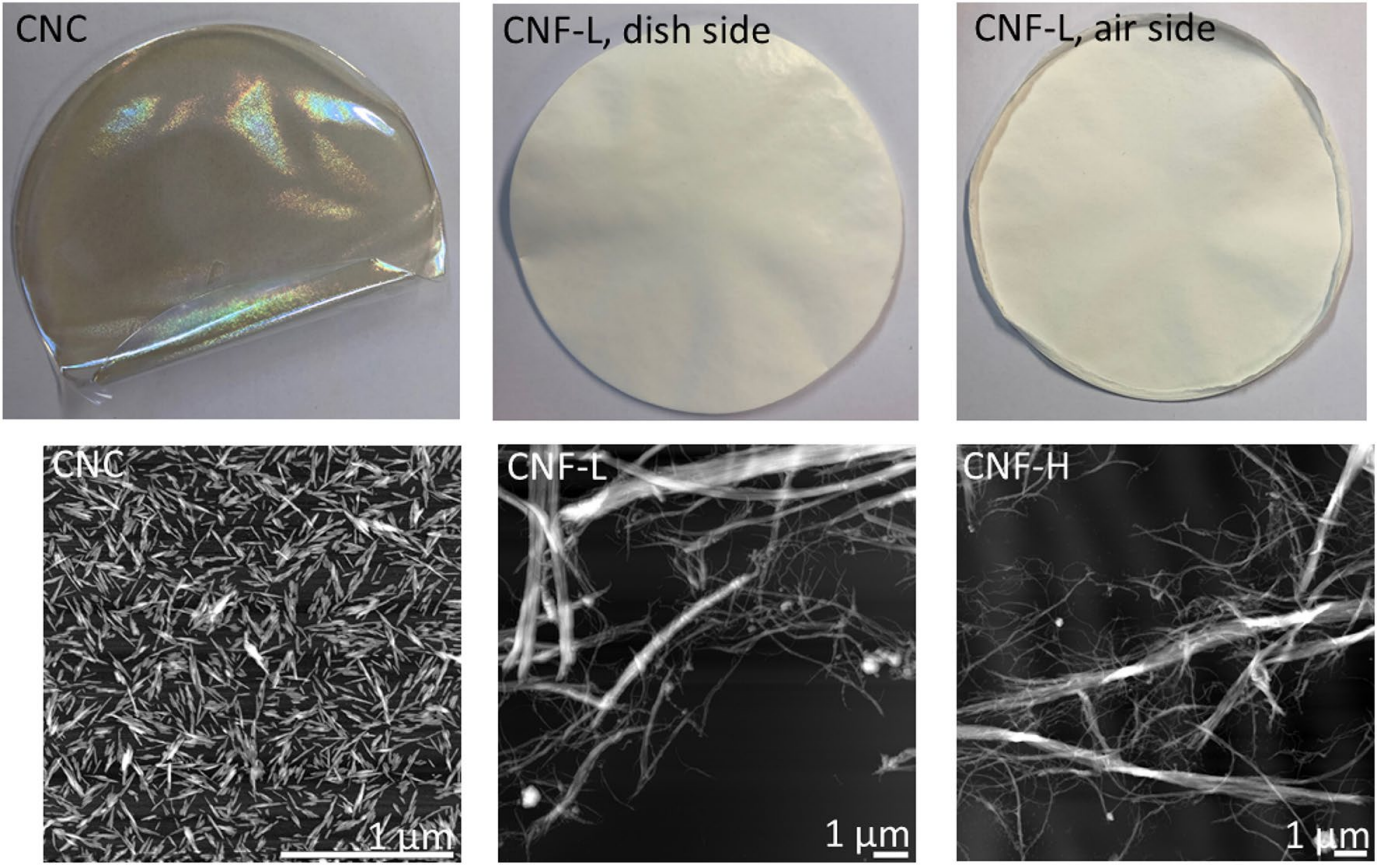
Nanocellulose films used for swelling tests in this paper. Bottom row: AFM micrographs. AFM of CNCs are adapted from Torstensen et al.^46^ (with premission from Elsevier).

The CNFs and CNCs employed in this paper have been previously characterized. A summary of the characterization is given in Table 2 and Fig. 3. CNCs are well characterized by Sacui et al.^7^, described as rods of the length of 100–200 nm and width of 6–7 nm. Similar dimensions were also verified by Torstensen et al.^46^. The CNCs have a crystalline fraction of 60% (determined by NMR)^7^. The CNFs have been characterized by Ottesen et al.^12^, and have a fibrillar shape and a width ≥ 3 nm and lengths in the nm—µm scale. The crystallinity of CNF-L is between 30 and 40%, while CNF-H is > 60% (determined by NMR and XRD).

**Table 2:** Summary of suspension properties and films employed in this paper.

Parameter	CNC	CNF-L	CNF-H
Charge (µmol/g)	~ 300^46^	~ 250^b^^47^	~ 250^b^^47^
Crystallinity (%)	60^7^	44+/− 2 (NMR) 32 (XRD)^12^	65+/− 2 (NMR) 69 (XRD)^12^
Fiber length (mm)^a^	0.7+/− 0.1	0.43+/− 0.01	0.44
Presence of total fines (%)^a^	93.4+/− 2.4	82.1+/− 0.2	81.8
Presence of secondary fines (%)^a^	90+/− 1.4	56.90+/− 0.02	57.7
Objects × 10^–3^/g^a^	0	1.5+/− 0.3	5+/− 2
Object length (mm)^a^	Not detected	0.4+/− 0.1	0.44+/− 0.05
Object width (mm)^a^	Not detected	0.27+/− 0.05	0.28+/− 0.02

CNF-L/H films were virtually indistinguishable. The error estimates are standard errors.

^a^Measured on nanocellulose suspension using Fibertester.

^b^Measured on the native pulp. No errors indicate that values were identical between parallels.

The macroscopic suspension morphology probed by Fibertester indicated that the CNF-L and CNF-H suspensions were similar. Residual fiber length was 0.4 mm, while fines were ~ 80% and secondary fines ~ 60%. However, the CNCs contained significantly more nanosized material and fewer larger pulp remnants. This observation is supported by the number of secondary fines in CNC, which was ~ 90% compared to ~ 60% in CNF-L/H. A higher presence of secondary fines indicates more nanocellulose in the suspension^48^. Moreover, the CNC suspensions contain close to zero objects, also suggesting that more of the suspension is nanosized^48^.

#### Thermal properties

The thermal properties were consistent with CNF/CNC materials.A detailed analysis of TGA and DSC profiles is provided in SI-1.

#### Film swelling and mechanical properties

Film swelling was investigated at 25 °C and 80%RH (state A), and then 39 °C and 36%RH (state B) (Table 3). These two states have a density of 0.018 kg water/m^3^ or ~ 2.5 kPa water vapour pressure. Swelling (S, Eq. 1) was ~ 7–8% at State A for both CNC and CNF-L/H films. We note that 7–8% swelling is similar to Belbekhouche et al.^17^ results of 13% of mechanical CNF films. When examining AFM, TGA, and DSC profiles, it should be evident that CNF-L/H films are essentially very similar. The nanocellulose morphology and thermal decomposition properties are interchangeable. However, they have a degree of crystallinity difference of ~ 20%. It is worthwhile to reflect on the crystallinity differences between CNF-H and -L. It is either 21% (XRD) or 37% (NMR) (found in Table 2). The difference in crystallinity corresponds to 0.21 g/g film or 0.37 g/g film of additional crystalline cellulose in CNF-H films. Our findings of similar swelling agree with the notion that higher relative humidities are required to observe differences between amorphous and crystalline cellulose^19^.

**Table 3:** Film swelling, S (Eq. 1), after t = 168 h at either state A or state B. Both states correspond to an absolute humidity of ~ 0.018 kg/m^3^ water.

Film type	State A (25 °C and 80%RH)	State B (39 °C and 36%RH)
CNC	7.9+/− 0.5	1.2+/− 0.2
CNF–L	7.6+/− 0.4	2.2+/− 0.4
CNF–H	7+/− 0.4	2+/− 0.2

The reported errors are standard errors.

The transition from state A to state B is marked by decreased swelling. Similar studies by Belbekhouche et al.^17^ (see Fig. 1) show that swelling at 80%RH at 25 °C is in the clustering regime of sorption. We note that a temperature increase of 14 °C is enough to reduce swelling to 1–2%. By a direct comparison (Fig. 3 in Belbekhouche et al.^17^), this modest temperature increase corresponds to a reduction in swelling values equivalent to reducing the relative humidity of about 70–75% (from 80% to below 10%RH) if all swelling experiments were conducted at ambient conditions. This highlights that temperature has a significant influence on swelling. The reason is most likely that the entropic gain of deswelling increases with temperature (ΔG = ΔH − TΔS). The reduced sorption at higher temperatures has been discussed by e. g. Nelson et al.^49^. Furthermore; swelling results can be interpreted considering Lindman's paper^50^. They discuss the enhanced solubility of cellulose that arises in polar solvent systems at lower temperatures. Several reasons for this behavior have been debated, and the leading opinion is that cellulose acquires a more hydrophobic conformation at elevated temperatures, thus hindering dissolution^51^. This should also lead to water desorption at higher temperatures if the amount of water present in the system is constant.

As all film types are essentially cellulose, the swelling should be comparable, with the discrepancies that CNCs may pack more densely due to their smaller dimensions. Denser packing may impede swelling, as observed by Torstensen et al.^52^ They found that CNF films cast at higher temperatures (in the range of 25–50 °C) had a more complex film structure with dense regions as well as macroscopic bubbles. Therefore, films cast at higher temperatures swelled less. The effect of film preparation on the accessible surface area has been highlighted by e. g. Torstensen et al., where drying methods were instrumental in controlling the film 3D morphology^53^. Moreover, increased nanocellulose charge increases swelling^54^. However, the native pulp for CNF production used in this study has a charge of^47^ ~ 250 µmol/g, while CNC had ~ 300 µmol/g (Table 2). Since the swelling of CNF/CNC in state A is similar, we reason that charge does not play a role in the water clustering regime. The slight difference between CNC/CNF swelling at state B is most likely due to a difference in monolayer sorption capacity. These differences could also be caused by the difference in charged group (sulfate ester in CNC, carboxylic acid in CNF) or film structural features, i. e. available area for sorption or chemical dissimilarities. Swelling in liquid water of CNF-L and –H nanocellulose fibrils has been reported as 44–45% and 30–34%, respectively^22^. This observed differences in liquid but not in vapour in this study is in agreement with the literature and is most likely due to differences in crystallinity caused by ammonia treatment.

To investigate if the material was stiffer in state A compared to state B, the strength of CNF film types was characterized (SI-2). This was not possible for CNC films due to the inherent brittleness of such films. In this study, the transition from swollen state A to state B was followed by a significant increase in the tensile index, namely 13%. For CNF-L this increase was (66.5+/− 1.6) kNm/kg to (79.6+/− 3.3) kNm/kg and for CNF-H the increase was from (61+/− 1) kNm/kg to (76+/− 3.3) kNm/kg. We note that the swelling ratios are ~ 7%/2% = 3.5 (state A/state B), while tensile index ratios were ~ 1.2 (state B/state A) for CNF-L / CNF-H films. No difference in elongation at the breaking point was detected.

### Part II: Molecular dynamics simulation of fibril swelling

The molecular modeling aims to understand the experimental observations of swelling better. In the experimental section, the swelling was investigated at a constant absolute humidity of ~ 0.018 kg water/m^3^. In this section, we study this process with constant water content in a fixed-volume simulation box (Fig. 2).

#### Fibril analysis before swelling

A detailed fibril morphological analysis is provided in SI-5. After energy minimization (EM), the result was a fibril with V_cellulose fibril_ = 354 nm^3^ (estimated by Delaunay triangulation) and a corresponding density of 1.4 g/cm^3^. The calculated density is close to literature values for cellulose^55^ (1.5–1.6 g/cm^3^), and the slight discrepancy should be considered an artefact of the nanometric size of the studied object. Moreover, determining fibril volume regardless of technique is associated with an error, discussed and reviewed by Connolly^56^. Volume inaccuracies may also be due to OPLS forcefield inaccuracies.

#### Water sorption at 25 °C

**Figure 4:**
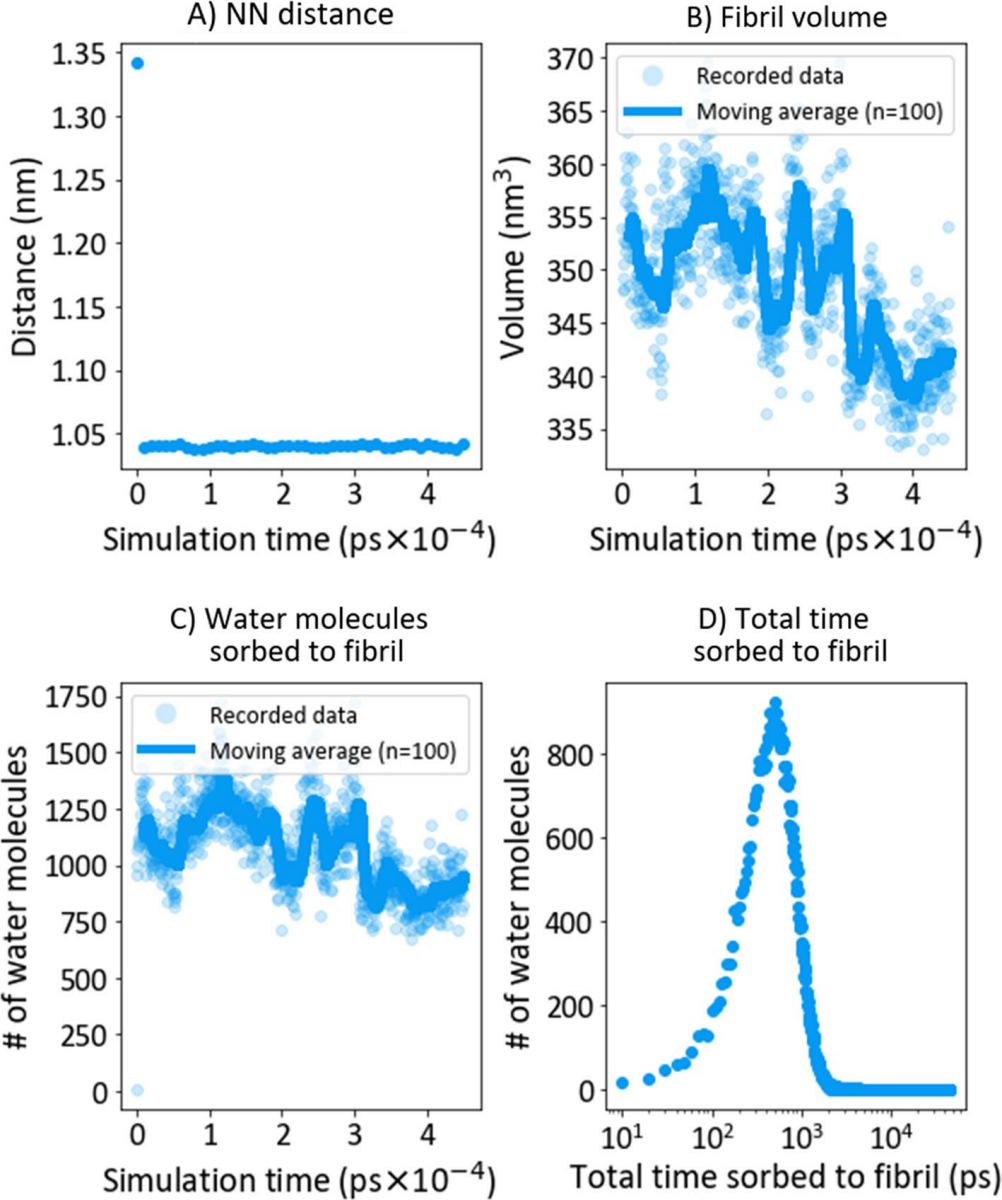
Swelling at 25 °C. (**A**) Nearest neighbour (NN) distance, (**B**) the bundle volume, (**C**) the number of sorbed water molecules, and (**D**) the total time water was sorbed to a fibril.

This section describes the swelling/water sorption process at 25 °C. The swelling was characterized by the 50 nearest neighbor (NN) water molecules of each carbon (Fig. 4A). The initial average nearest-neighbour (NN) distance was about 1.3 nm due to the initial placing of water molecules in the simulation setup. The average value decreased from 1.3 nm to a threshold of ~ 1 nm after the first 10 ps. The NN-distance reached equilibrium at this point. No volumetric expansion was detected during swelling (Fig. 4B). Fibril volumetric expansion was found by Ottesen et al.^22^ They measured the fibril swelling by AFM to be 34% and 44% for CNF-H and -L, respectively. The mean number of sorbed water molecules in this study was (1+/− 0.2) × 10^3^(Fig. 4C), corresponding to S = (1000 × 18 g mol^−1^/6.023 × 10^23^ mol^−1^)/5.06 × 10^–19^ g ~ 7% (Eq. 1). This value is far from water liquid swelling of film and fibrils, except for studies by Aulin et al.^57^ finding between 7 and 26% swelling for films in liquid water. We note first and foremost that the modeled system is single fibril, where Ottesen et al.^22^ is a reasonable comparison (30–40% swelling of a single fibril). We reason that swelling is reduced since our system has a density below that of liquid water. The mean total sorption time of water was (692+/− 639) or approximately 1.53% of the total simulation time (Fig. 4D).

**Figure 5:**
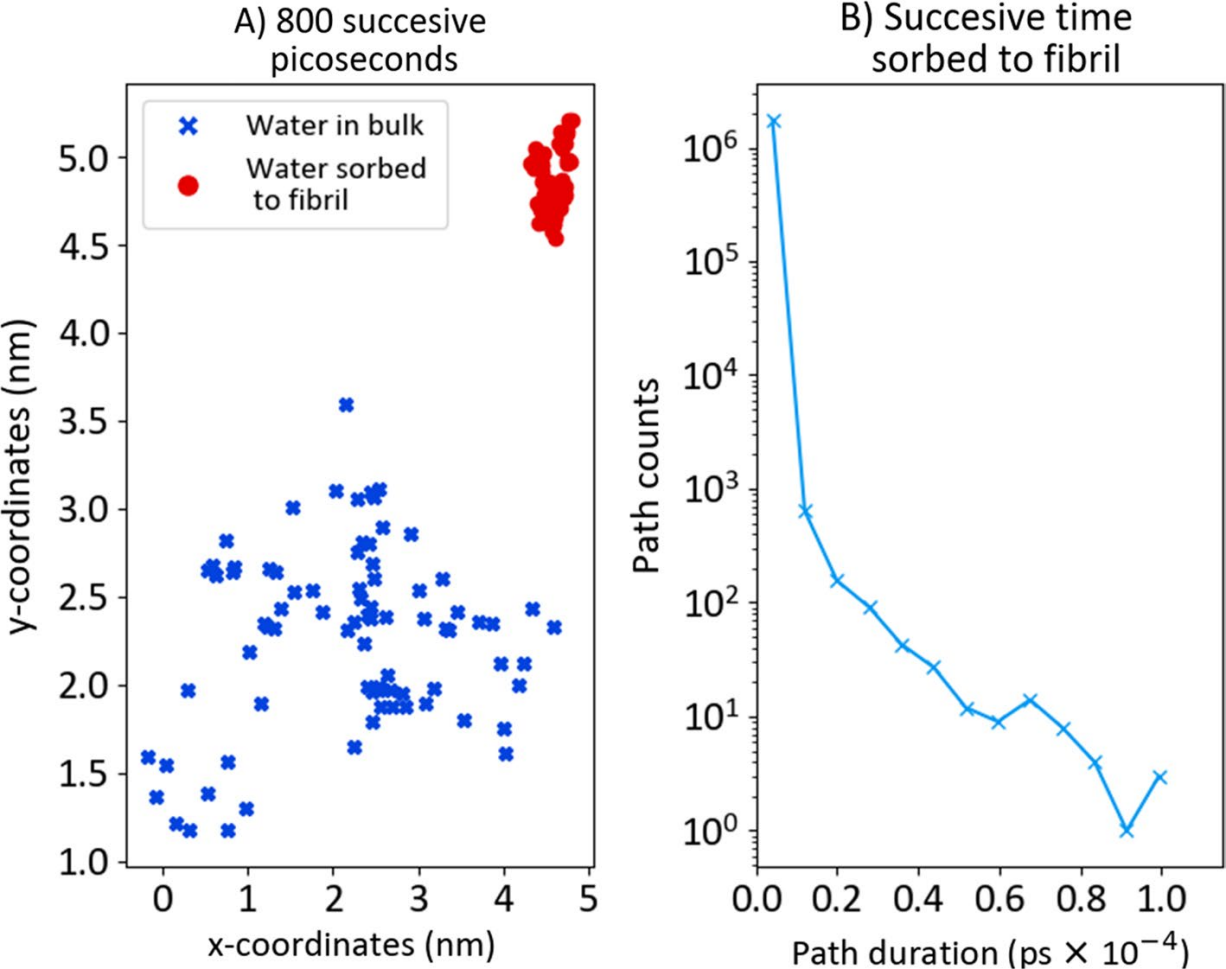
(**A**) Successive 800 ps (80 steps) diffusion in the bulk and sorbed to the fibril of the same water molecule. (**B**) Successive time a water molecule spends sorbed to a fibril. Simulations were performed at 25 °C.

To understand the phenomenological difference between water diffusion in the bulk and water sorbed to the fibril, the diffusion of the same water molecule is plotted for successive 800 ps in the bulk and sorbed to the bundle (Fig. 5A). Clearly, water molecules experienced trapping when sorbed to the bundle. A water molecule could be in the bulk and sorbed during the simulation. Of interest was the distribution of continuous time that a water molecule spent sorbed to a fibril before desorbing to the bulk (Fig. 5B).

**Figure 6:**
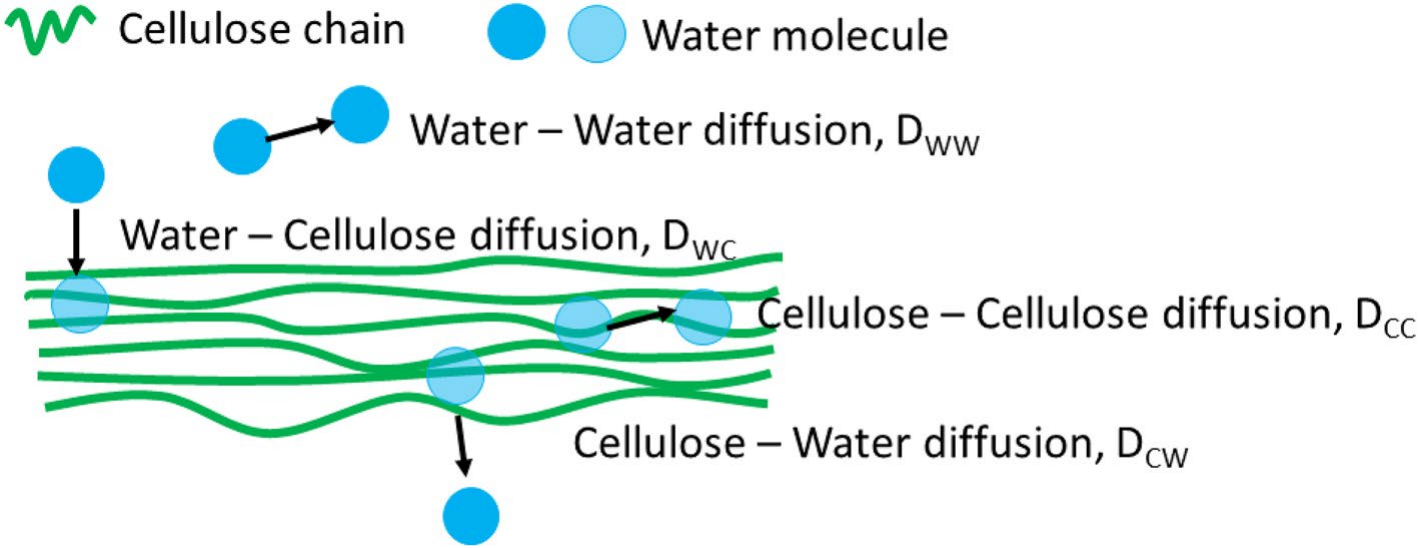
The different phases for analyzing the diffusion of water molecules.

Based on the observations in Fig. 5, water-trapping occurred when molecules were sorbed to the fibril. This observed trapping was analyzed by investigating the diffusion coefficient. The method for determining the diffusion coefficient (D) is explained in SI-6 and was based on the equation:

2 $${\mathrm{MSD}}_{\mathrm{P}}(\mathrm{t})=\sum_{1}^{{\mathrm{n}}_{\mathrm{P}}}{\left({\mathrm{x}}_{\mathrm{t}+10}-{\mathrm{x}}_{\mathrm{t}}\right)}^{2}+{\left({\mathrm{y}}_{\mathrm{t}+10}-{\mathrm{y}}_{\mathrm{t}}\right)}^{2}+ {\left({\mathrm{z}}_{\mathrm{t}+10}-{\mathrm{z}}_{\mathrm{t}}\right)}^{2} = 6{\mathrm{D}}_{\mathrm{P}}{\mathrm{t}}_{\mathrm{tot},\mathrm{P}},$$

where the interval $(\mathrm{t},\mathrm{ t}+10)$ is one step with $\mathrm{\Delta t}\hspace{0.17em}$= 10 ps, and where n_P_ is the total number of steps experienced by all water molecules in each phase (P). The total time in each phase was t_tot,P._ This method was verified against other methods of calculating D. The steps of diffusion in one phase were recorded. The diffusion coefficient in that phase, D_P_ was calculated by the method "From all measurements with two intervals", where the two intervals were (0, t_tot,P_/2) and ﻿(t_tot,P_/2, ﻿t_tot,P_) respectively. Phases corresponded to water molecule diffusion in the bulk water phase (WW) diffusion, sorbed to the cellulose bundle (CC) or across the interphase (either WC or CW). Diffusion phases are illustrated in Fig. 6. We determined the type of diffusion by the pointLocation-function in Matlab.

Diffusion in bulk water was D_WW_ = (3.7000+/− 0.0014) × 10^–5^ cm^2^/s. The bulk diffusion was comparable to water diffusion in a water system without a fibril, D_WWneat_ = (3.6000+/− 0.0017) × 10^–5^ cm^2^/s. Moreover, there was a clear decrease in D from water to the fibril, with D_CW/WC_ ~ 2.2 × 10^–5^ cm^2^/s and D_CC_ ~ 0.65 × 10^–5^ cm^2^/s. Reduced diffusion coefficients at boundaries and cellulose indicated that water molecules were "stuck" to the fibril and strongly sorbed to cellulose. Detailed calculations are given in SI-7.

#### Water sorption at elevated temperatures

**Figure 7:**
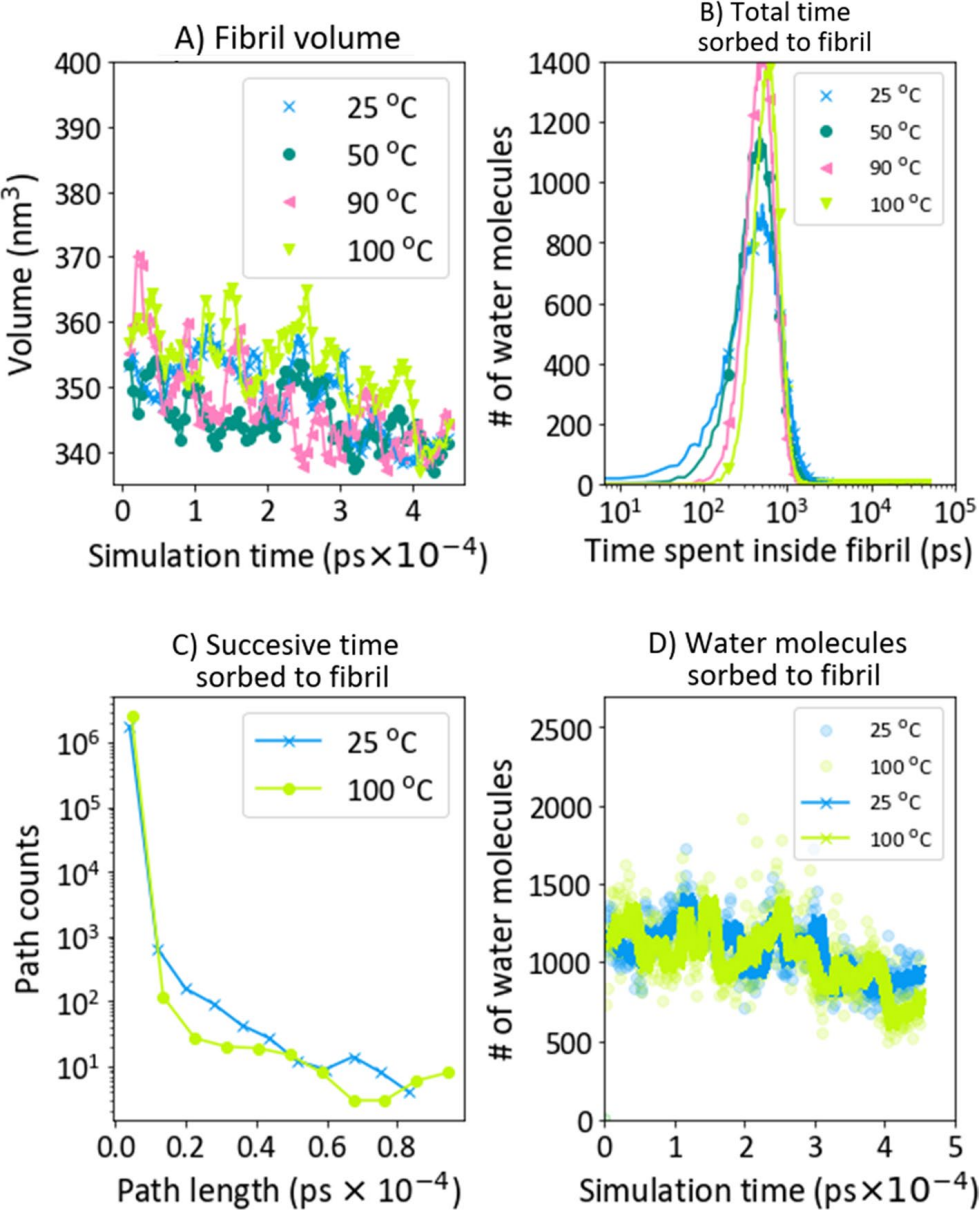
Swelling at different temperatures. Curves in (**A**) and (**D**) represent a moving average with n = 100. In (**D**), points are actual measurements.

Methods in the previous section analyzed the swelling at elevated temperatures. The nearest neighbour distance did not change with increasing swelling temperature (SI-8). Neither did the fibril volume (Fig. 7A). The fluctuations in volume and atom coordinates did not allow for a detailed examination of the bundle chain conformation. The total time a water molecule spent sorbed to the fibril is shown in Fig. 7B (the results for 25 °C are also shown in Fig. 5D). There was a clear peak narrowing with increasing temperatures. A more in-depth analysis is given in SI-8. A reduction was also observed from 25 °C to 100 °C in the continuous path distribution [Fig. 7C (25 °C also in Fig. 5B)]. It is worthwhile to reflect on the difference in swelling from 25 to 100 °C. Water molecules spend on average the same time sorbed in these temperatures. However, due to kinetic fluctuations, water molecules are more continuously sorbed to the fibril at lower temperatures. The number of water molecules sorbed to the fibril was independent of temperature (Fig. 7D). Given the lower density (803 kg/m^3^) of the water phase compared to actual TIP4P density (above 900 kg/m^3^ in the entire investigated temperature regime^44^), we would expect desorption of water from the bundle. The reason for not detecting this is at present not known.

**Figure 8:**
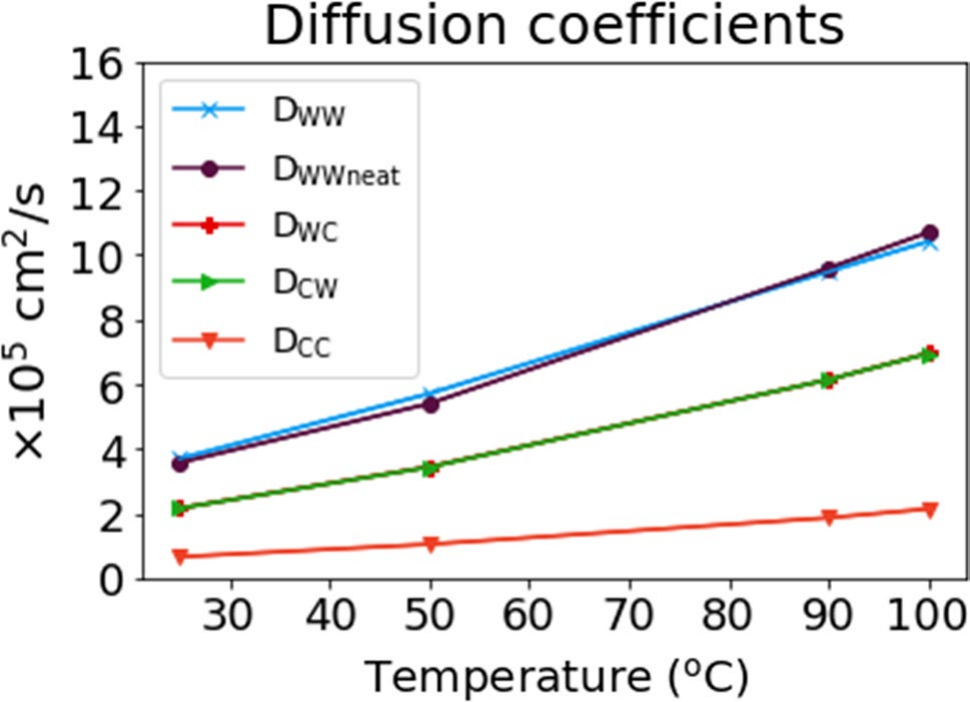
Diffusion coefficients in bulk water (D_WW_) and for water molecules sorbed to the cellulose bundle (D_CC_). The diffusion in the bulk phase was validated against the diffusion of water calculated by Gromacs (Gromacs_MSD, ref. 8 in SI, Eq.1 in SI-6) in a system without a cellulose bundle (D_WWneat_, described in SI-6).

Another possible hypothesis was that water was more evenly distributed in the fibril at lower temperatures and that water was located more to the fibril surface at higher temperatures. This would agree with water molecules spending less successive time sorbed to a fibril at higher temperatures. The distribution of sorbed water was investigated at different temperatures (S1-9). Water ordering could not be verified as the minimal water displacement between frames was 0.2–0.4 nm. This "resolution" is too low to distinguish if water was on the fibril surface or inside the fibril. The diffusion constant was calculated for water molecules in different parts of the water/fibril system (Fig. 8). Water diffusion for neat water (as described in SI-6) and the bulk water in the water/fibril system (D_WW_) were compared to validate the modeling results. These values were in close agreement, thus validating the chosen method (Eq. 2). The diffusion in water was D_WW,25_ was (3.7000 ± 0.0014) × 10^–5^ cm^2^/s, while D_WW,100_ was (10.4 ± 0.003) × 10^–5^ cm^2^/s However, diffusion of water molecules sorbed to the cellulose bundle (D_CC_) was reduced compared to D_WW_ at all temperatures. D_CC,25_ was (0.65 ± 0.0042) × 10^–5^ cm^2^/s, while D_CC,100_ was (2.1 ± 0.02) × 10^–5^ cm^2^/s. Moreover, this reduction was more pronounced at higher temperatures with D_WW,25_ − D_CC_, 25 ~ 3 × 10^–5^ cm/s^2^ at 25 °C, while D_WW,100_ − D_CC,100_ ~ 8.3 × 10^–5^ cm/s^2^. Diffusion of the TIP4P water model used is discussed by Rozmanov et al.^58^. They found D at about 3 × 10^–5^ cm^2^/s (0.3 Å^2^/ps) in ambient conditions, in agreement with our data. In general, linearity is found for elevated temperatures (above ambient), and diffusion is underestimated above 270 K, e. g. not following the traditional Arrhenius relationship above this temperature. From 280 to 310 K (7–37 °C), they found the activation energy of diffusion to be 17.1 kJ/mol. By fitting ln(D) = D_o_exp(E_A_/RT) in our entire range we found E_A_ = 14.7 kJ/mol. This supports the notion that D is underestimated at higher temperatures. Linear regression gave D_WW_(T) = 1.3 + 0.09 × T and D_CC_(T) = 0.1 + 0.02 × T, where T is the temperature in °C. We note here that the slope is 4.5 × higher in bulk water self-diffusion and that the temperature dependence of D is not as prominent compared to water sorbed to cellulose. Comparable values experimentally determined for water diffusion in cellulose^59^ are 10^–6^ to 10^–7^ cm^2^/s below 263 K (− 10 °C). They also determined the water diffusion coefficient to be 10^–6^ cm^2^/s at temperatures below 263 K (− 10 °C).

Restricted water self-diffusion in cellulose is in good agreement with others^60^. Restricted diffusion of water absorbed into cellulose is typically related to material pore sizes, and the diffusion constant is reduced if the path is larger than the pore size. However, in our case, most water molecules probably diffuse along the cellulose surface. Cellulose is believed to restrict diffusion physically and through cellulose-water molecular interactions. These restrictions also exist at increasing temperatures, disrupting the otherwise linear increase in diffusion for the TIP4P model.

### Ethics approval

The authors confirm that there were no ethical conflicts in preparing this manuscript.

### Consent to participate

All authors consent to participate in this work.

## Conclusions

In this paper, we have investigated the swelling of nanocellulose films by swelling experiments and molecular dynamics. We find that temperature significantly influences swelling compared to relative humidity. At fixed absolute humidity of 0.018 kg/m^3^, increasing the temperature by 14 °C, from 25 to 39 °C corresponds to reducing swelling of CNC/CNF films from 7 to 2%. This result underlines temperature as a highly efficient way of controlling cellulosic material swelling and possibly counteracting the detrimental effects of subjecting cellulosic materials to high relative humidity. Moreover, we find partial evidence of reduced sorption in molecular modeling. Water molecules spend less time inside the fibril between 25 to 100 °C. However, we did not observe desorption in our experiments. We investigated the diffusion coefficient of water in the bulk and bound to cellulose. The diffusion in bulk water (D_WW_) was D_WW,25_ = (3.7000 ± 0.0014) × 10^–5^ cm^2^/s, while D_WW,100_ = (10.4 ± 0.003) × 10^–5^ cm^2^/s However, diffusion of water molecules sorbed to the cellulose bundle (D_CC_) was reduced compared to D_WW_ at all temperatures. The diffusion constant of water sorbed to cellulose at 25 °C, was D_CC,25_ = (0.65 ± 0.0042) × 10^–5^ cm^2^/s, while D_CC,100_ = (2.1 ± 0.02) × 10^–5^ cm^2^/s. Our results confirm that modeling supports deswelling experiments. However, no gravimetric deswelling is observed in modeling, even after a large increase in temperature. More work is needed to understand why water sorbed to cellulose experiences a smaller diffusion coefficient increase than bulk water as temperature increases.

## Supplementary Information

Supplementary Information.

## Data Availability

Modeling scripts are given in the supporting information.
