# Supplementary material for: The influence of temperature on cellulose swelling at constant water density
Source: Sci Rep. 2022 Dec 1;12:20736. doi: 10.1038/s41598-022-22092-5 (PMC9715729; doi:10.1038/s41598-022-22092-5)
Supplement: Supplementary file 1 — Supplementary Information. [file 41598_2022_22092_MOESM1_ESM.docx]

­­­­The influence of temperature on cellulose swelling at constant water density

Jonathan Torstensen^a)*^ (0000-0002-8632-8028), Vegar Ottensen^b)^ (0000-0003-1810-2710), Sandra Rodríguez-Fabià^c)^ (0000-0002-4273-231X), Kristin Syverud ^b,c)^ (0000-0003-2271-3637), Lars Johansson^c)^ (0000-0001-5522-0009), Anders Lervik^d)^ (0000-0002-1505-6731)

1. Western Norway University of Applied Sciences, Bergen, Norway
2. Department of Chemical Engineering, NTNU, Trondheim, Norway
3. RISE PFI, Trondheim, Norway
4. Department of Chemistry, NTNU, Trondheim, Norway

* Corresponding author, [jonathan.torstensen@hvl.no](mailto:jonathan.torstensen@hvl.no)

**SUPPORTING INFORMATION**

**SI – 1: Decomposition analysis**

Differential scanning calorimetry (DSC) and thermogravimetric analysis (TGA) were performed to discern nanocellulose thermal properties.  Tests were performed in nitrogen and air (**Table S1, Fig. S1**).

Thermogravimetric analysis in nitrogen indicated no significant difference in decomposition between CNF-L and CNF-H samples, with a degradation onset above 300 ^o^C. This is in agreement with the decomposition of (ligno)cellulosic biomasses, with cellulose and lignin degradation between 300 ^o^C – 400 ^o^C, and hemicellulose degradation from 150 ^o^C to 300 ^o^C.^1,2^ Cotton linters are typically regarded as close to pure cellulose, and a decomposition temperature above 300 ^o^C is thus expected. Conversely, CNC-type films displayed faster degradation and contained more residual carbon. CNCs are considered to be close to pure cellulose,

due to the sulfuric acid hydrolysis process's removal of lignin and hemicellulose. The earlier onset of degradation in CNC films may thus be explained by the presence of sulfate half-esters, introduced by the hydrolysis treatment. The presence of sulfate esters typically results in faster degradation kinetics, as discussed by Lu et al.^3^ Differential scanning calorimetry in nitrogen indicated that the crystallinity was indeed more elevated in CNF-H compared to CNF-L samples. There was evidence of higher degradation energy, with ΔE ˜ 20 J/g increase in CNF-H compared to CNF-L. Degradation of CNC required less energy, again assumed to be due to the higher sulfuric ester content. ^3^

Thermal degradation in air is a combustion type of reaction. This was markedly different from thermal degradation in nitrogen for CNC, CNF-L, and –H type of samples. While decomposition in nitrogen occurred over a narrow temperature range, there was a marked plateau in air decomposition. This plateau was indicative of slower reaction kinetics. Slower kinetics were assumed to be caused by a more diverse gas composition, i. e. 100 % N_2_ vs. ~ 78/21 % N_2_/O_2_.

Moreover, air-treated samples were close to completely degraded with very little residual mass. Air DSC profiles of CNF-L/-H indicated a more complex, two-phase degradation process with two endothermic peaks. Moreover, CNC displayed one small exothermic peak before a more prominent endothermic peak.

***Table S1.****DSC and TGA of films in nitrogen and air. The onset temperature of degradation and residual mass is extrapolated from TGA curves (*), while the degradation energy is extrapolated from DSC curves (**). The error estimates are standard errors*

| Key parameters | Onset of degradation (^o^C) * | Residual mass at 1000 ^o^C (%) * | Energy of degradation (J/g) ** |
| --- | --- | --- | --- |
| Nitrogen | | | |
| CNC | 260.0 +/- 0.2 | 28.2 +/- 0.2 | 69.1 +/- 0.2 |
| CNF- L | 342.8 +/- 0.4 | 9.5 +/- 0.2 | 179 +/- 7 |
| CNF – H | 335.1 +/- 0.5 | 9.3 +/- 0.5 | 232 +/- 5 |
| Air | | | |
| CNC | 261.0 +/- 0.3 | 3.0 +/- 0.5 | 6180 +/- 50 |
| CNF – L | 326.0 +/- 0.5 | 1. +/- 0.2 | 1285 +/- 60 |
| CNF – H | 321 +/- 0.2 | 0.6 +/- 0.2 | 1105 +/-32 |

**
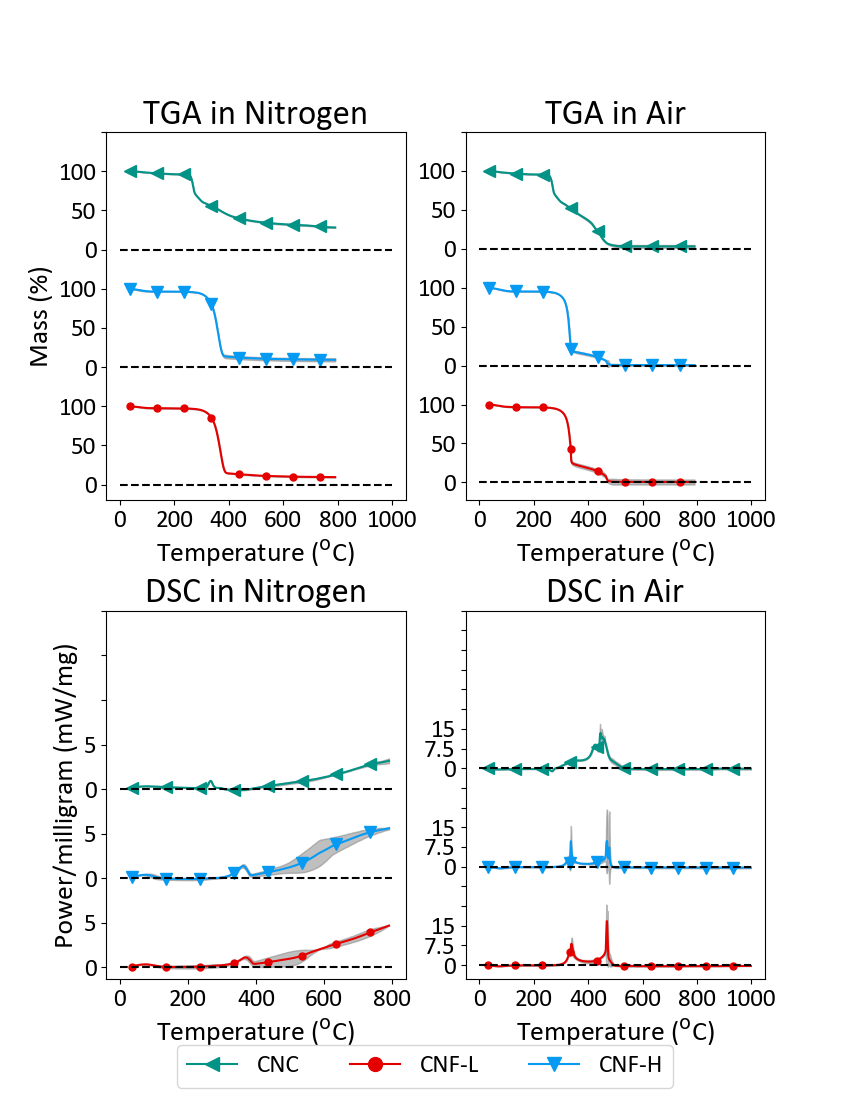
**

***Figure S1.****DSC and TGA of films in nitrogen and air. Graphs represent the average interpolation of two samples. The gray area represents the difference between interpolations*

**SI-2: Mechanical properties**

Mechanical properties of CNF films (tensile index, strain at break) did not vary due to crystallinity, in partial agreement with Ottesen et al.^4^ They found an increase in Young's modulus with increasing crystallinity but no significant changes in the strain at break. In this study, the transition from swollen state A to state B was followed by a significant increase in the tensile index, namely 13 %. For CNF-L this increase was (66.5 +/- 1.6) kNm/kg to (79.6 +/- 3.3) kNm/kg and for CNF-H the increase was from (61 +/- 1) kNm/kg to (76 +/- 3.3) kNm/kg. This transition has been described by others, where a decline in Young's modulus and tensile strength has been found with increasing film water uptake.^5^ We note that the swelling ratios are ~ 7%/2% = 3.5 (state A/state B), while tensile index ratios were ~1.2 (state B/state A). Data are provided in **Figure S2**.

***Table S2.*** *Stress/strain behaviour of CNF-L and CNF-H films at different states.*

| State A (25 ^o^C and 80 %RH) | | |
| --- | --- | --- |
| Film type | Tensile index (kNm/kg) | Elongation at break (%) |
| CNF – L (S = 7.6 +/- 0.4) | 66.5 +/- 1.6 | 5.3 +/- 0.1 |
| CNF – H (S = 7 +/- 0.4) | 61 +/- 1 | 7.6 +/- 1 |
| State B (39 ^o^C and 36 %RH) | | |
| CNF – L (S = 2.2 +/- 0.4) | 79.6 +/- 3.3 | 4.4 +/- 0.5 |
| CNF – H (S = 2 +/- 0.2) | 76 +/- 3.3 | 6.8 +/- 0.6 |


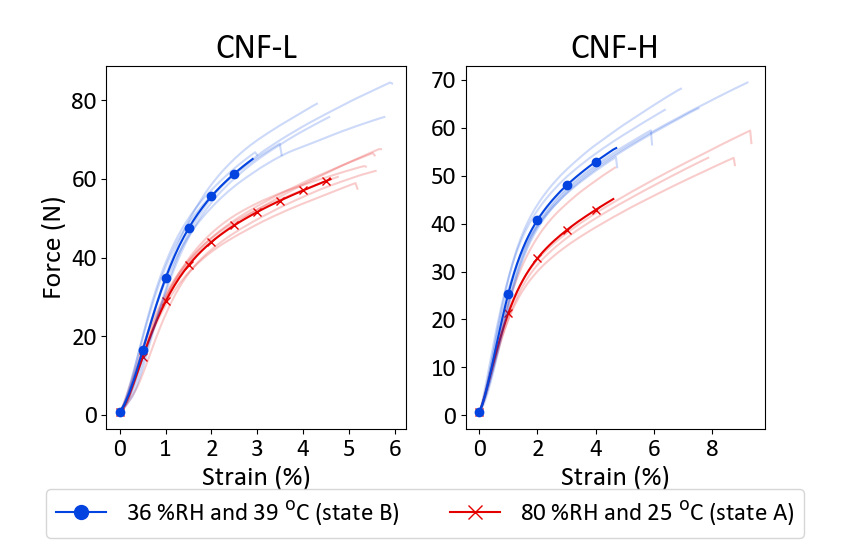


***Figure S2.*** *Stress/strain behaviour of CNF-L and CNF-H films at different states. For CNF-L, n=6 and for CNF-H n = 4 in state A, while n = 6 (state B). Opaque lines are actual measurements, while the presented main curves are the average of interpolated curves*

**SI-3: NPT (Energy minimization, EM)**

;

; VARIOUS PREPROCESSING OPTIONS

; Preprocessor information: use cpp syntax.

; e.g.: -I/home/joe/doe -I/home/mary/roe

include =

; e.g.: -DPOSRES -DFLEXIBLE (note these variable names are case sensitive)

define =

; RUN CONTROL PARAMETERS

integrator = steep

; Start time and timestep in ps

tinit = 0

dt = 0.001

nsteps = 1000000

; For exact run continuation or redoing part of a run

init-step = 0

; Part index is updated automatically on checkpointing (keeps files separate)

simulation-part = 1

; mode for center of mass motion removal

comm-mode = Linear

; number of steps for center of mass motion removal

nstcomm = 100

; group(s) for center of mass motion removal

comm-grps =

; LANGEVIN DYNAMICS OPTIONS

; Friction coefficient (amu/ps) and random seed

bd-fric = 0

ld-seed = -1

; ENERGY MINIMIZATION OPTIONS

; Force tolerance and initial step-size

emtol = 10

emstep = 0.01

; Max number of iterations in relax-shells

niter = 20

; Step size (ps^2) for minimization of flexible constraints

fcstep = 0

; Frequency of steepest descents steps when doing CG

nstcgsteep = 1000

nbfgscorr = 10

; TEST PARTICLE INSERTION OPTIONS

rtpi = 0.05

; OUTPUT CONTROL OPTIONS

; Output frequency for coords (x), velocities (v) and forces (f)

nstxout = 0

nstvout = 0

nstfout = 0

; Output frequency for energies to log file and energy file

nstlog = 10

nstcalcenergy = 10

nstenergy = 10

; Output frequency and precision for .xtc file

nstxout-compressed = 1000

compressed-x-precision = 1000

; This selects the subset of atoms for the compressed

; trajectory file. You can select multiple groups. By

; default, all atoms will be written.

compressed-x-grps = System

; Selection of energy groups

energygrps =

; NEIGHBORSEARCHING PARAMETERS

; cut-off scheme (Verlet: particle based cut-offs, group: using charge groups)

cutoff-scheme = Verlet

; nblist update frequency

nstlist = 10

; ns algorithm (simple or grid)

ns_type = grid

; Periodic boundary conditions: xyz, no, xy

pbc = xyz

periodic-molecules = no

; Allowed energy error due to the Verlet buffer in kJ/mol/ps per atom,

; a value of -1 means: use rlist

verlet-buffer-tolerance = 0.005

; nblist cut-off

rlist = 1

; long-range cut-off for switched potentials

rlistlong = -1

nstcalclr = -1

; OPTIONS FOR ELECTROSTATICS AND VDW

; Method for doing electrostatics

coulombtype = PME

coulomb-modifier = Potential-shift-Verlet

rcoulomb-switch = 0

rcoulomb = 1.0

; Relative dielectric constant for the medium and the reaction field

epsilon-r = 1

epsilon-rf = 0

; Method for doing Van der Waals

vdw-type = Cut-off

vdw-modifier = Potential-shift-Verlet

; cut-off lengths

rvdw-switch = 0

rvdw = 1.0

; Apply long range dispersion corrections for Energy and Pressure

DispCorr = EnerPres

; Extension of the potential lookup tables beyond the cut-off

table-extension = 1

; Separate tables between energy group pairs

energygrp-table =

; Spacing for the PME/PPPM FFT grid

fourierspacing = 0.16

; FFT grid size, when a value is 0 fourierspacing will be used

fourier-nx = 0

fourier-ny = 0

fourier-nz = 0

; EWALD/PME/PPPM parameters

pme_order = 4

ewald-rtol = 1e-05

ewald-rtol-lj = 0.001

lj-pme-comb-rule = Geometric

ewald-geometry = 3d

epsilon-surface = 0

; IMPLICIT SOLVENT ALGORITHM

implicit-solvent = No

; GENERALIZED BORN ELECTROSTATICS

; Algorithm for calculating Born radii

gb-algorithm = Still

; Frequency of calculating the Born radii inside rlist

nstgbradii = 1

; Cutoff for Born radii calculation; the contribution from atoms

; between rlist and rgbradii is updated every nstlist steps

rgbradii = 1

; Dielectric coefficient of the implicit solvent

gb-epsilon-solvent = 80

; Salt concentration in M for Generalized Born models

gb-saltconc = 0

; Scaling factors used in the OBC GB model. Default values are OBC(II)

gb-obc-alpha = 1

gb-obc-beta = 0.8

gb-obc-gamma = 4.85

gb-dielectric-offset = 0.009

sa-algorithm = Ace-approximation

; Surface tension (kJ/mol/nm^2) for the SA (nonpolar surface) part of GBSA

; The value -1 will set default value for Still/HCT/OBC GB-models.

sa-surface-tension = -1

; OPTIONS FOR WEAK COUPLING ALGORITHMS

; Temperature coupling

tcoupl = V-rescale

nsttcouple = -1

nh-chain-length = 10

print-nose-hoover-chain-variables = no

; Groups to couple separately

tc-grps = system

; Time constant (ps) and reference temperature (K)

; ENDRE TEMP HER:

tau_t = 0.5

ref_t = 298.15

; pressure coupling

pcoupl = no; Parrinello-Rahman

pcoupltype = isotropic

nstpcouple = -1

; Time constant (ps), compressibility (1/bar) and reference P (bar)

tau_p = 2.0

compressibility = 4.5e-5

ref_p = 1.0

; Scaling of reference coordinates, No, All or COM

refcoord-scaling = No

; OPTIONS FOR QMMM calculations

QMMM = no

; Groups treated Quantum Mechanically

QMMM-grps =

; QM method

QMmethod =

; QMMM scheme

QMMMscheme = normal

; QM basisset

QMbasis =

; QM charge

QMcharge =

; QM multiplicity

QMmult =

; Surface Hopping

SH =

; CAS space options

CASorbitals =

CASelectrons =

SAon =

SAoff =

SAsteps =

; Scale factor for MM charges

MMChargeScaleFactor = 1

; Optimization of QM subsystem

bOPT =

bTS =

; SIMULATED ANNEALING

; Type of annealing for each temperature group (no/single/periodic)

annealing =

; Number of time points to use for specifying annealing in each group

annealing-npoints =

; List of times at the annealing points for each group

annealing-time =

; Temp. at each annealing point, for each group.

annealing-temp =

; GENERATE VELOCITIES FOR STARTUP RUN

gen_vel = yes

; ENDRE TEMP HER:

gen_temp = 298.15

gen-seed = -1

; OPTIONS FOR BONDS

constraints = all-bonds

; Type of constraint algorithm

constraint_algorithm = lincs

; Do not constrain the start configuration

continuation = no

; Use successive overrelaxation to reduce the number of shake iterations

Shake-SOR = no

; Relative tolerance of shake

shake-tol = 0.0001

; Highest order in the expansion of the constraint coupling matrix

lincs_order = 4

; Number of iterations in the final step of LINCS. 1 is fine for

; normal simulations, but use 2 to conserve energy in NVE runs.

; For energy minimization with constraints it should be 4 to 8.

lincs_iter = 1

; Lincs will write a warning to the stderr if in one step a bond

; rotates over more degrees than

lincs-warnangle = 30

; Convert harmonic bonds to morse potentials

morse = no

; ENERGY GROUP EXCLUSIONS

; Pairs of energy groups for which all non-bonded interactions are excluded

energygrp-excl =

; WALLS

; Number of walls, type, atom types, densities and box-z scale factor for Ewald

nwall = 0

wall-type = 9-3

wall-r-linpot = -1

wall-atomtype =

wall-density =

wall-ewald-zfac = 3

; COM PULLING

pull = no

; ENFORCED ROTATION

; Enforced rotation: No or Yes

rotation = no

; Group to display and/or manipulate in interactive MD session

IMD-group =

; NMR refinement stuff

; Distance restraints type: No, Simple or Ensemble

disre = No

; Force weighting of pairs in one distance restraint: Conservative or Equal

disre-weighting = Conservative

; Use sqrt of the time averaged times the instantaneous violation

disre-mixed = no

disre-fc = 1000

disre-tau = 0

; Output frequency for pair distances to energy file

nstdisreout = 100

; Orientation restraints: No or Yes

orire = no

; Orientation restraints force constant and tau for time averaging

orire-fc = 0

orire-tau = 0

orire-fitgrp =

; Output frequency for trace(SD) and S to energy file

nstorireout = 100

; Free energy variables

free-energy = no

couple-moltype =

couple-lambda0 = vdw-q

couple-lambda1 = vdw-q

couple-intramol = no

init-lambda = -1

init-lambda-state = -1

delta-lambda = 0

nstdhdl = 50

fep-lambdas =

mass-lambdas =

coul-lambdas =

vdw-lambdas =

bonded-lambdas =

restraint-lambdas =

temperature-lambdas =

calc-lambda-neighbors = 1

init-lambda-weights =

dhdl-print-energy = no

sc-alpha = 0

sc-power = 1

sc-r-power = 6

sc-sigma = 0.3

sc-coul = no

separate-dhdl-file = yes

dhdl-derivatives = yes

dh_hist_size = 0

dh_hist_spacing = 0.1

; Non-equilibrium MD stuff

acc-grps =

accelerate =

freezegrps =

freezedim =

cos-acceleration = 0

deform =

; simulated tempering variables

simulated-tempering = no

simulated-tempering-scaling = geometric

sim-temp-low = 300

sim-temp-high = 300

; Electric fields

; Format is number of terms (int) and for all terms an amplitude (real)

; and a phase angle (real)

E-x =

; Time dependent (pulsed) electric field. Format is omega, time for pulse

; peak, and sigma (width) for pulse. Sigma = 0 removes pulse, leaving

; the field to be a cosine function.

E-xt =

E-y =

E-yt =

E-z =

E-zt =

; Ion/water position swapping for computational electrophysiology setups

; Swap positions along direction: no, X, Y, Z

swapcoords = no

; AdResS parameters

adress = no

; User defined thingies

user1-grps =

user2-grps =

userint1 = 0

userint2 = 0

userint3 = 0

userint4 = 0

userreal1 = 0

userreal2 = 0

userreal3 = 0

userreal4 = 0

**SI-4: NVT (45 ns Molecular Dynamics, MD)**

; VARIOUS PREPROCESSING OPTIONS

; Preprocessor information: use cpp syntax.

; e.g.: -I/home/joe/doe -I/home/mary/roe

include =

; e.g.: -DPOSRES -DFLEXIBLE (note these variable names are case sensitive)

define =

; RUN CONTROL PARAMETERS

integrator = md

; Start time and timestep in ps

tinit = 0

dt = 0.001

nsteps = 4000000

; For exact run continuation or redoing part of a run

init-step = 0

; Part index is updated automatically on checkpointing (keeps files separate)

simulation-part = 1

; mode for center of mass motion removal

comm-mode = Linear

; number of steps for center of mass motion removal

nstcomm = 100

; group(s) for center of mass motion removal

comm-grps =

; LANGEVIN DYNAMICS OPTIONS

; Friction coefficient (amu/ps) and random seed

bd-fric = 0

ld-seed = -1

; ENERGY MINIMIZATION OPTIONS

; Force tolerance and initial step-size

emtol = 10

emstep = 0.01

; Max number of iterations in relax-shells

niter = 20

; Step size (ps^2) for minimization of flexible constraints

fcstep = 0

; Frequency of steepest descents steps when doing CG

nstcgsteep = 1000

nbfgscorr = 10

; TEST PARTICLE INSERTION OPTIONS

rtpi = 0.05

; OUTPUT CONTROL OPTIONS

; Output frequency for coords (x), velocities (v) and forces (f)

nstxout = 0

nstvout = 0

nstfout = 0

; Output frequency for energies to log file and energy file

nstlog = 10

nstcalcenergy = 10

nstenergy = 10

; Output frequency and precision for .xtc file

nstxout-compressed = 1000

compressed-x-precision = 1000

; This selects the subset of atoms for the compressed

; trajectory file. You can select multiple groups. By

; default, all atoms will be written.

compressed-x-grps = System

; Selection of energy groups

energygrps =

; NEIGHBORSEARCHING PARAMETERS

; cut-off scheme (Verlet: particle based cut-offs, group: using charge groups)

cutoff-scheme = Verlet

; nblist update frequency

nstlist = 10

; ns algorithm (simple or grid)

ns_type = grid

; Periodic boundary conditions: xyz, no, xy

pbc = xyz

periodic-molecules = no

; Allowed energy error due to the Verlet buffer in kJ/mol/ps per atom,

; a value of -1 means: use rlist

verlet-buffer-tolerance = 0.005

; nblist cut-off

rlist = 1

; long-range cut-off for switched potentials

rlistlong = -1

nstcalclr = -1

; OPTIONS FOR ELECTROSTATICS AND VDW

; Method for doing electrostatics

coulombtype = PME

coulomb-modifier = Potential-shift-Verlet

rcoulomb-switch = 0

rcoulomb = 1.0

; Relative dielectric constant for the medium and the reaction field

epsilon-r = 1

epsilon-rf = 0

; Method for doing Van der Waals

vdw-type = Cut-off

vdw-modifier = Potential-shift-Verlet

; cut-off lengths

rvdw-switch = 0

rvdw = 1.0

; Apply long range dispersion corrections for Energy and Pressure

DispCorr = EnerPres

; Extension of the potential lookup tables beyond the cut-off

table-extension = 1

; Separate tables between energy group pairs

energygrp-table =

; Spacing for the PME/PPPM FFT grid

fourierspacing = 0.16

; FFT grid size, when a value is 0 fourierspacing will be used

fourier-nx = 0

fourier-ny = 0

fourier-nz = 0

; EWALD/PME/PPPM parameters

pme_order = 4

ewald-rtol = 1e-05

ewald-rtol-lj = 0.001

lj-pme-comb-rule = Geometric

ewald-geometry = 3d

epsilon-surface = 0

; IMPLICIT SOLVENT ALGORITHM

implicit-solvent = No

; GENERALIZED BORN ELECTROSTATICS

; Algorithm for calculating Born radii

gb-algorithm = Still

; Frequency of calculating the Born radii inside rlist

nstgbradii = 1

; Cutoff for Born radii calculation; the contribution from atoms

; between rlist and rgbradii is updated every nstlist steps

rgbradii = 1

; Dielectric coefficient of the implicit solvent

gb-epsilon-solvent = 80

; Salt concentration in M for Generalized Born models

gb-saltconc = 0

; Scaling factors used in the OBC GB model. Default values are OBC(II)

gb-obc-alpha = 1

gb-obc-beta = 0.8

gb-obc-gamma = 4.85

gb-dielectric-offset = 0.009

sa-algorithm = Ace-approximation

; Surface tension (kJ/mol/nm^2) for the SA (nonpolar surface) part of GBSA

; The value -1 will set default value for Still/HCT/OBC GB-models.

sa-surface-tension = -1

; OPTIONS FOR WEAK COUPLING ALGORITHMS

; Temperature coupling

tcoupl = V-rescale

nsttcouple = -1

nh-chain-length = 10

print-nose-hoover-chain-variables = no

; Groups to couple separately

tc-grps = system

; Time constant (ps) and reference temperature (K)

; ENDRE TEMP HER:

tau_t = 0.5

ref_t = 298.15

; pressure coupling

pcoupl = no

pcoupltype = isotropic

nstpcouple = -1

; Time constant (ps), compressibility (1/bar) and reference P (bar)

tau_p = 2.0

compressibility = 4.5e-5

ref_p = 1.0

; Scaling of reference coordinates, No, All or COM

refcoord-scaling = No

; OPTIONS FOR QMMM calculations

QMMM = no

; Groups treated Quantum Mechanically

QMMM-grps =

; QM method

QMmethod =

; QMMM scheme

QMMMscheme = normal

; QM basisset

QMbasis =

; QM charge

QMcharge =

; QM multiplicity

QMmult =

; Surface Hopping

SH =

; CAS space options

CASorbitals =

CASelectrons =

SAon =

SAoff =

SAsteps =

; Scale factor for MM charges

MMChargeScaleFactor = 1

; Optimization of QM subsystem

bOPT =

bTS =

; SIMULATED ANNEALING

; Type of annealing for each temperature group (no/single/periodic)

annealing =

; Number of time points to use for specifying annealing in each group

annealing-npoints =

; List of times at the annealing points for each group

annealing-time =

; Temp. at each annealing point, for each group.

annealing-temp =

; GENERATE VELOCITIES FOR STARTUP RUN

gen_vel = yes

; ENDRE TEMP HER:

gen_temp = 298.15

gen-seed = -1

; OPTIONS FOR BONDS

constraints = all-bonds

; Type of constraint algorithm

constraint_algorithm = lincs

; Do not constrain the start configuration

continuation = no

; Use successive overrelaxation to reduce the number of shake iterations

Shake-SOR = no

; Relative tolerance of shake

shake-tol = 0.0001

; Highest order in the expansion of the constraint coupling matrix

lincs_order = 4

; Number of iterations in the final step of LINCS. 1 is fine for

; normal simulations, but use 2 to conserve energy in NVE runs.

; For energy minimization with constraints it should be 4 to 8.

lincs_iter = 1

; Lincs will write a warning to the stderr if in one step a bond

; rotates over more degrees than

lincs-warnangle = 30

; Convert harmonic bonds to morse potentials

morse = no

; ENERGY GROUP EXCLUSIONS

; Pairs of energy groups for which all non-bonded interactions are excluded

energygrp-excl =

; WALLS

; Number of walls, type, atom types, densities and box-z scale factor for Ewald

nwall = 0

wall-type = 9-3

wall-r-linpot = -1

wall-atomtype =

wall-density =

wall-ewald-zfac = 3

; COM PULLING

pull = no

; ENFORCED ROTATION

; Enforced rotation: No or Yes

rotation = no

; Group to display and/or manipulate in interactive MD session

IMD-group =

; NMR refinement stuff

; Distance restraints type: No, Simple or Ensemble

disre = No

; Force weighting of pairs in one distance restraint: Conservative or Equal

disre-weighting = Conservative

; Use sqrt of the time averaged times the instantaneous violation

disre-mixed = no

disre-fc = 1000

disre-tau = 0

; Output frequency for pair distances to energy file

nstdisreout = 100

; Orientation restraints: No or Yes

orire = no

; Orientation restraints force constant and tau for time averaging

orire-fc = 0

orire-tau = 0

orire-fitgrp =

; Output frequency for trace(SD) and S to energy file

nstorireout = 100

; Free energy variables

free-energy = no

couple-moltype =

couple-lambda0 = vdw-q

couple-lambda1 = vdw-q

couple-intramol = no

init-lambda = -1

init-lambda-state = -1

delta-lambda = 0

nstdhdl = 50

fep-lambdas =

mass-lambdas =

coul-lambdas =

vdw-lambdas =

bonded-lambdas =

restraint-lambdas =

temperature-lambdas =

calc-lambda-neighbors = 1

init-lambda-weights =

dhdl-print-energy = no

sc-alpha = 0

sc-power = 1

sc-r-power = 6

sc-sigma = 0.3

sc-coul = no

separate-dhdl-file = yes

dhdl-derivatives = yes

dh_hist_size = 0

dh_hist_spacing = 0.1

; Non-equilibrium MD stuff

acc-grps =

accelerate =

freezegrps =

freezedim =

cos-acceleration = 0

deform =

; simulated tempering variables

simulated-tempering = no

simulated-tempering-scaling = geometric

sim-temp-low = 300

sim-temp-high = 300

; Electric fields

; Format is number of terms (int) and for all terms an amplitude (real)

; and a phase angle (real)

E-x =

; Time dependent (pulsed) electric field. Format is omega, time for pulse

; peak, and sigma (width) for pulse. Sigma = 0 removes pulse, leaving

; the field to be a cosine function.

E-xt =

E-y =

E-yt =

E-z =

E-zt =

; Ion/water position swapping for computational electrophysiology setups

; Swap positions along direction: no, X, Y, Z

swapcoords = no

; AdResS parameters

adress = no

; User defined thingies

user1-grps =

user2-grps =

userint1 = 0

userint2 = 0

userint3 = 0

userint4 = 0

userreal1 = 0

userreal2 = 0

userreal3 = 0

userreal4 = 0

**SI-5: Fibril analysis**

The fibril (**Fig. S3**) was divided into 52 "levels" corresponding to each monomer. The initial fibril length was 26.9 nm. Each level thus consisted of 216 (6×36) carbon atoms. Fibril cross-section examination indicated that this was approximately square. Cross sections had an area of (12.7 +/- 0.13) nm^2^. Assuming a perfectly square shape (A = s^2^), gave a side length of (3.6 +/- 0.02) nm. Side lengths agree with a cellulose elementary fibril width of ̃2-5 nm measured by AFM.^6,7^ At this point, we emphasize that the current perception is a 6 by 3 chain geometry in the native fibril. However, native fibril / nanocellulose agglomeration and difference in the number of chains between species are believed to be why larger fibril widths are measured experimentally. By calculating the distance between the first C4 to the last C1 for each chain, the new fibril length was estimated to be 26.2 nm +/- 0.1 nm. The reduced length corresponded to a decrease of $100(1- \frac{26.2 nm}{26.9 nm})=2.6 \%$ shrinking in the length direction. The extension for each chain was calculated as: distance(first C4, last C1)/26.9 nm, and an extension map of all chains indicated that they were about fully extended. The least extended chains were typically located at the fibril surface.

**
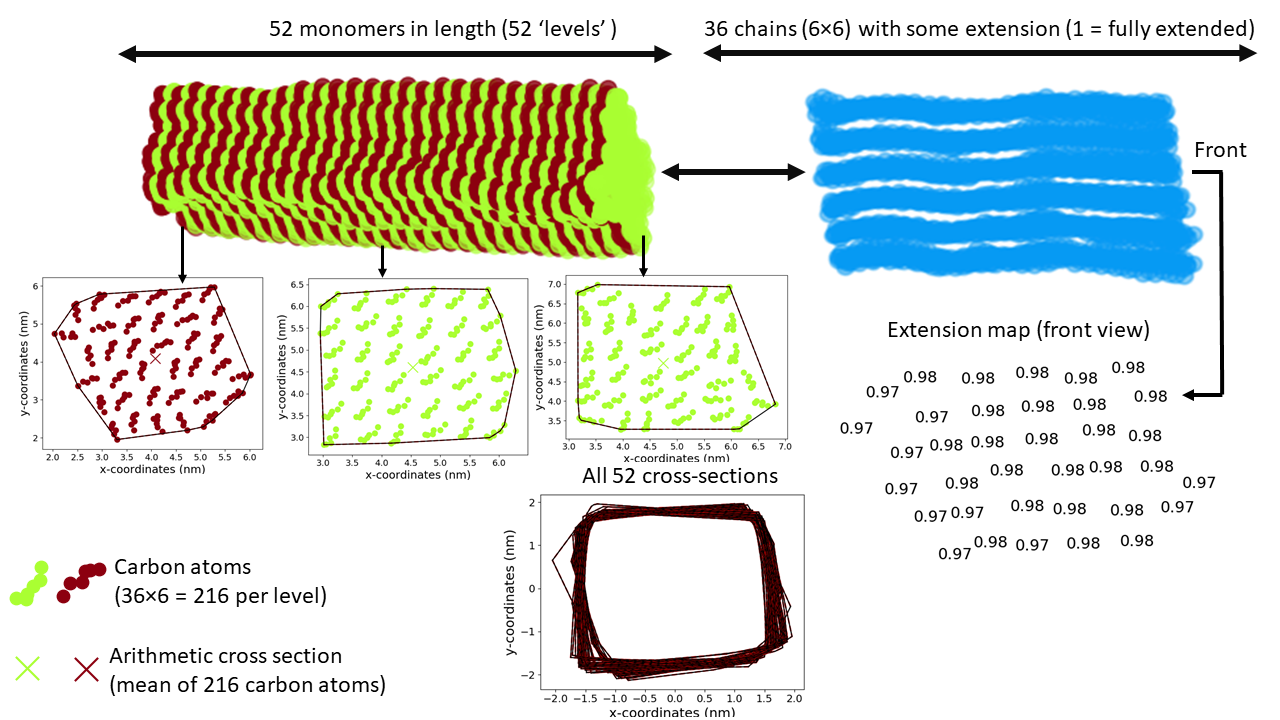
**

***Figure S3.*** *Fibril formation and analysis. A) The initial Fig. 1, with dimensions and angles. The monomer length was found by dividing the chain length by the number of monomers, by the width was taken as the maximum length between different atom pairs. B) Cross-sections revealed a predominantly square shape with rounded corners. Analysis of chain – extensions revealed that the least extended chains were located along the fibril edges and corners.*

**SI-6: Diffusion constant calculations**

Calculating the diffusion constant may be done by several methods. In Gromacs,^8^ the mean-square-displacement (MSD) is plotted against t, and the diffusion constant is found by regression:

$\underset{}{MSD=\frac{1}{N}\sum_{1}^{N} {\lim_{t\to\infty} {(r}_{t}-r_{0})}^{2}} =6Dt$ (1),

where *r_0_* is the starting position, *r_t_* is the position at a given time *t*, *N* is the number of molecules, and *D is* the diffusion constant. The MSD is calculated as the average over all atoms, and regression errors is the difference between D in the first and second half of the interval.

Water molecule movement in our system is more complex as it includes: movement in the water phase, sorbed to the fibril, and across the boundaries (Main text, **Figure 4**). As the chosen time-step was 10 ps, all types of diffusion may occur during one step. However, this effect was assumed to have minimal effect on the results. Each water molecule (represented by the oxygen atom) trajectory was used to calculate the MSD as:

$\mathrm{MS}D_{i}(t)=\sum_{1}^{n_{p,i}} \left( x_{t+10}-x_{t} \right)^{2}+\left( y_{t+10}-y_{t} \right)^{2}+ \left( z_{t+10}-z_{t} \right)^{2} = 6Dt,$(2)

where the interval $(t, t+10)$ is one step with $\Delta t$ = 10 ps, and where n_p_ is the total number of diffusion events experienced by each water molecule (i) in a given phase (as in **Figure 6**).

Different methods of employing **Equation 2**, were examined in a neat 1728 water (without a fibril) molecule system diffusing for 100 steps (1000 ps) at 25 ^o^C (**Table S3**). This corresponds to the WW phase. It was clear that while the average D is ~ 3.6×10^-5^ cm^2^/s, and that it is possible to tailor/tune its deviation. Based on this, the method "From all measurements with two intervals" was chosen, having an acceptable computational cost while also producing an error estimate.

**Table S3**. The neat water diffusion coefficient, *D* (cm^2^/s) at 25 ^o^C.

| **Method** | **D (cm^2^/s)** | **Comment** |
| --- | --- | --- |
| Gromacs 5.1.4 | (3.6 +/- 0.0017)× 10^-5^ | Method GMX _MSD^8^ |
| From each molecule | (3.6 +/- 0.3)× 10^-5^ | Regression of MSD (**Eq. 2**) from for t = 0 to t = 1000 ps for each molecule. The diffusion constant is the average value. The error is the standard deviation. |
| From all measurements with two intervals | (3.6+/-0.0015)× 10^-5^ | All molecules are treated as one, and all observations (1728 molecules x 100 steps) are divided into two intervals ( t = 0-500, t = 500-1000). Regression of MSD (**Eq. 2**) is employed. The diffusion constant is the average of the two values. The error is the difference between the obtained D values. |
| From all measurements without intervals | (3.6)× 10^-5^ | All molecules are treated as one, and all observations (1728 molecules x 100 steps) are used. Regression of MSD (**Eq. 2**) is employed. |
| Average of displacement between frames | (3.6+/-3.2)× 10^-5^ | The average displacement between frames for all water molecules is used:  ${D=(\left( x_{t+10}-x_{t} \right)}^{2}+\left( y_{t+10}-y_{t} \right)^{2}+ \left( z_{t+10}-z_{t} \right)^{2}$)/6Δt,  Where Δt = 10 ps.  The error is the standard deviation. |

**SI – 7: Diffusion constant calculations at 25 ^o^C**

***Table S4.*** *Water molecule diffusion constants calculated by* ***Eq. 2****, with the method "From all measurements with two intervals" in* ***Table S3****. The total number of events was 4500 (number of steps) × 69545 (number of water molecules) = 312952500*

| Diffusion-type | D (cm^2^/s, **Eq. 2**) | Number of events (sum of all n_p,i_ in **Eq. 2**) | Experienced by number of water molecules |
| --- | --- | --- | --- |
| Water-Water (D_WW_) | (3.7000+/-0.0014) × 10^-5^ | 306377899 (97.8 %) | 69545 |
| Water-Cellulose (D_WC_) | (2.2000+/-0.0061) × 10^-5^ | 1761976 (0.6 %) | 69530 |
| Cellulose-Water (D_CW_) | (2.2000+/-0.0086) × 10^-5^ | 1761094 (0.6 %) | 69530 |
| Cellulose-Cellulose (D_CC_) | (0.65+/-0.0042) × 10^-5^ | 3051531 (1 %) | 69342 |
| SUM |  | 312952500 (100%) |  |

**SI-8: Water sorption in cellulose**

***Table S4.*** *Properties of water sorption in cellulose at different temperatures.*

| Temperature (^o^C) | Total time spent inside the fibril, all water molecules (ps/10^9^) | Mean total time per water molecule (ps) (+/- standard deviation) | Mode – time (ps) | Full width half maximum (ps) (**Fig. 7B**) |
| --- | --- | --- | --- | --- |
| 25 | 0.048 | 692 +/- 639 | 510 | 709 |
| 50 | 0.042 | 604 +/- 445 | 480 | 545 |
| 90 | 0.040 | 577 +/- 369 | 480 | 444 |
| 100 | 0.046 | 668 +/- 547 | 640 | 444 |

**
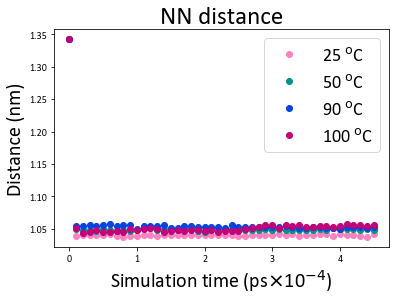
**

***Figure S4****. Nearest neighbour (NN) distance at different temperatures.*

**SI – 9: Surface water analysis**

There was some evidence that ordering of water molecules occurred as the simulation progressed, and there was some indication that water was more located to the surface at 100 ^o^C compared to 25 ^o^C. To evaluate this further, the carbon atoms in the fibril were manipulated to become one line of beads (**Fig. SI-5A)**. As discussed, the fibril was 36 chains, each consisting of 52 glucose monomers. Thus, there are 216 (6×36) carbon atoms per 'level' (see **Fig.** **S3**). Each of these levels was approximated by the mean, and the nearest distance from the line to each water molecule was detected (illustrated in SI 5). There was no phenomenological difference in sorption between 25 ^o^C and 100 ^o^C

The shortest distance between each sorbed water molecule to the string of beads was then calculated, and the average distance is represented in **Fig. SI-5B.** The average distance from the center was close to 2 nm in both cases. The diffusion between frames at 25 ^o^C was $s_{\mathrm{diff}}=\sqrt{\left( 6*D_{cc,25}*\Delta t \right)}= \sqrt{\left( 6*0.7*{10}^{-5}*\frac{{10}^{14}nm^{2}}{s}*10*{10}^{-12}s= \right)} =0.2 nm$

This corresponds to the average water molecule displacement between frames, sorbed to cellulose at 25 ^o^C. The same calculation at 100 ^o^C, gave s_diff_ = 0.4 nm. The magnitude of this length indicated that water molecule movement between frames was significant enough to affect the results. Considering a fibril with a width of 3.7 nm, it is not feasible to determine exactly where the water molecule is located – at the surface or in the fibril interior.

This was reflected in an average standard deviation of 0.3 nm in these measurements. Moreover, examining the distance of water molecules from the center at different times (**Fig. S5C-D**), strengthened the notion that the chosen time-step (10 ps) is too large to investigate if the position of water molecules inside or at the surface of a fibril. It is thus concluded that sorption includes both adsorption and absorption.


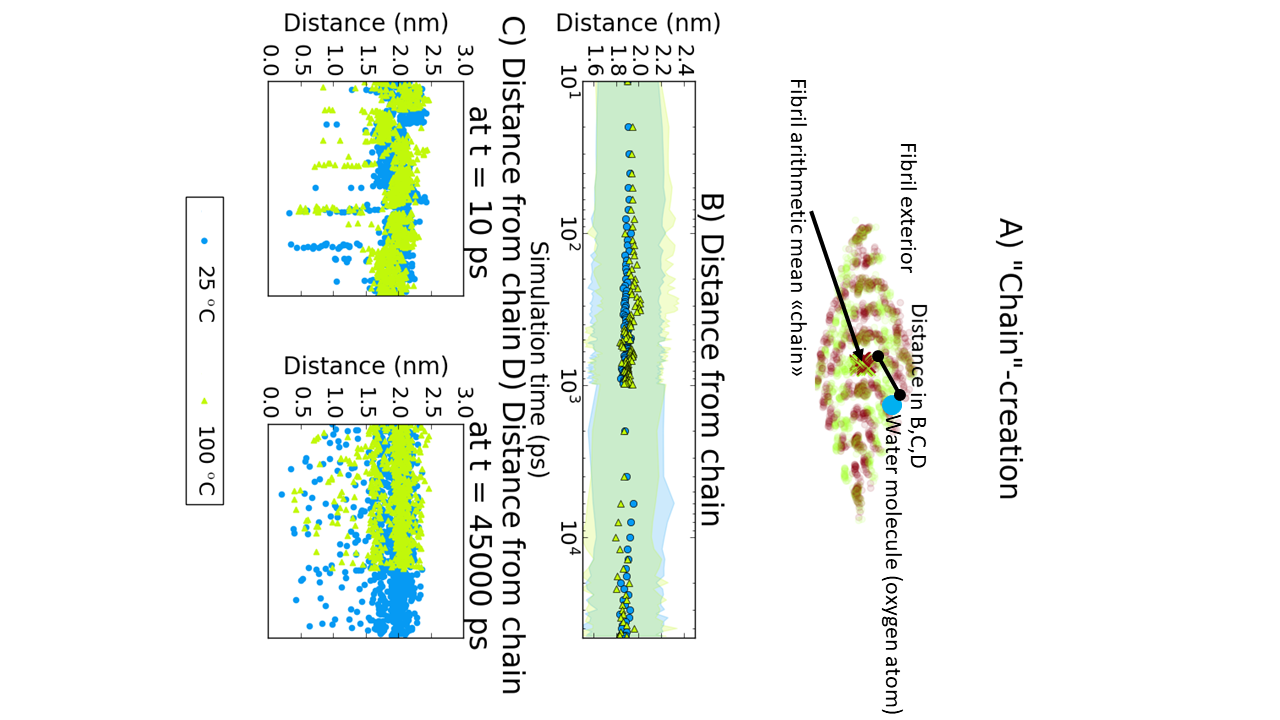


***Figure S5.*** *Water distribution at different times. A) The distance was calculated between sorbed water and a chain of each level arithmetic mean The standard deviation was 0.3 regardless of temperature. C-D) Each point represents a water molecule (represented by the water molecule oxygen atom)*

**References**

1. Caballero, J. A., Conesa, J. A., Font, R. & Marcilla, A. Pyrolysis kinetics of almond shells and olive stones considering their organic fractions. *J. Anal. Appl. Pyrolysis* **42**, 159–175 (1997).

2. Sanchez-Silva, L., López-González, D., Villaseñor, J., Sánchez, P. & Valverde, J. L. Thermogravimetric-mass spectrometric analysis of lignocellulosic and marine biomass pyrolysis. *Bioresour. Technol.* **109**, 163–172 (2012).

3. Lu, P. & Hsieh, Y. Lo. Preparation and properties of cellulose nanocrystals: Rods, spheres, and network. *Carbohydr. Polym.* **82**, 329–336 (2010).

4. Ottesen, V., Larsson, P. T., Chinga-Carrasco, G., Syverud, K. & Gregersen, Ø. W. Mechanical properties of cellulose nanofibril films: effects of crystallinity and its modification by treatment with liquid anhydrous ammonia. *Cellulose* **26**, 6615–6627 (2019).

5. Zhang, X., Yu, Y., Jiang, Z. & Wang, H. Influence of thickness and moisture content on the mechanical properties of microfibrillated cellulose (MFC) films. *Wood Res.* **61**, 851–860 (2016).

6. Ding, S. Y., Zhao, S. & Zeng, Y. Size, shape, and arrangement of native cellulose fibrils in maize cell walls. *Cellulose* **21**, 863–871 (2014).

7. Dufresne, A. *Nanocellulose: from nature to high performance tailored materials*. (De Gruyter, 2012).

8. GROMACS 2019 documentation. gmx msd. http://manual.gromacs.org/documentation/2019/onlinehelp/gmx-msd.html#gmx-msd.
